# Extension of the PRISMA 2020 statement for living systematic reviews (PRISMA-LSR): checklist and explanation

**DOI:** 10.1136/bmj-2024-079183

**Published:** 2024-11-19

**Authors:** Elie A Akl, Joanne Khabsa, Claire Iannizzi, Vanessa Piechotta, Lara A Kahale, James M Barker, Joanne E McKenzie, Matthew J Page, Nicole Skoetz, PRISMA-LSR Group, Tony Aburrow, Sabina Alam, Lisa Askie, Vivienne C Bachelet, Asma Ben Brahem, Lex Bouter, Isabelle Boutron, Romina Brignardello-Petersen, Jacob Burns, Anna Chaimani, Christine Chang, Stephanie Chang, Mike Clarke, Tammy Clifford, Kerry Dwan, Julian Elliott, Robin Featherstone, Markus Follmann, Christian Gluud, Gordon Guyatt, Lotty Hooft, Anna Jacobs, Svenja Krebs, Tamara Kredo, Toby Lasserson, Elizabeth Loder, Helen Macdonald, Iain Marshall, Steve McDonald, Matthew DF McInnes, Joerg Meerpohl, Maria-Inti Metzendorf, David Moher, Lorenzo Moja, Karel GM Moons, Mohammad Hassan Murad, Reem Mustafa, Stefano Negrini, Robby Nieuwlaat, Adriani Nikolakopoulou, Monika Nothacker, Rose O'Dea, Amir Qaseem, Gabriel Rada, Melissa L Rethlefsen, Ludovic Reveiz, Maria Ximena Rojas-Reyes, Jean-Paul Salameh, Georgia Salanti, Ian J Saldanha, Ashrita Saran, Jochen Schmitt, Holger J Schünemann, Richard Sever, Mark Simmonds, Bhagteshwar Singh, Anneliese Synnot, Britta Tendal Jeppesen, James Thomas, David Tovey, Andrea Tricco, Tari Turner, Per Olav Vandvik, Helen Wakeford, Stephanie Weibel, Vivian Welch, Wojtek Wiercioch

**Affiliations:** 1 Department of Internal Medicine, American University of Beirut, Beirut, Lebanon; 2 Department of Health Research Methods, Evidence, and Impact, McMaster University, Hamilton, Ontario, Canada; 3 Clinical Research Institute, American University of Beirut Medical Center, Beirut, Lebanon; 4 Institute of Public Health, Faculty of Medicine and University Hospital Cologne, University of Cologne, Cologne, Germany; 5 Immunization Unit, Department of Infectious Disease Epidemiology, Robert Koch-Institut, Berlin, Germany; 6 Infectious Disease Society of America, Arlington, VA, USA; 7 F1000 Research, London, UK; 8 Methods in Evidence Synthesis Unit, School of Public Health and Preventive Medicine, Monash University, Melbourne, Australia

## Abstract

Publications of living systematic reviews (LSRs) are increasing rapidly. Guidance facilitating transparent, complete, and accurate reporting of LSRs is needed. This paper reports the development of an extension of the Preferred Reporting Items for Systematic reviews and Meta-Analyses (PRISMA) 2020 statement for LSRs (PRISMA-LSR). The PRISMA-LSR extension includes the PRISMA-LSR checklist, the PRISMA-LSR flow diagram, reporting recommendations for the LSR status, and an explanation and elaboration document. This extension has been developed as an “add-on” to the PRISMA 2020 statement, meaning it should be used in addition to the PRISMA 2020 statement. The PRISMA-LSR extension is expected to benefit authors, editors, peer reviewers, and users of LSRs through transparent, complete, and accurate reporting of LSRs.

Living systematic reviews (LSRs) are attracting attention from researchers and medical journals.^1^ ^2^ Between 2014 (when the LSR approach first emerged^3^) and 2019, the rate of publication of these systematic reviews was low; however, since 2019, there has been a rapid increase. Indeed, the total number of LSRs published in 2020 and 2021 exceeded the total number published before 2020.^4^ LSRs are characterised by a continual search of the literature so that new evidence can be incorporated soon after it becomes available.^3^ ^5^ These reviews are particularly important when research is published rapidly and where concomitant policy decisions are required, such as during the covid-19 pandemic.^6^ ^7^ ^8^

The Preferred Reporting Items for Systematic reviews and Meta-Analyses (PRISMA) 2020 statement is intended to facilitate transparent, complete, and accurate reporting of systematic reviews.^9^ ^10^ ^11^ ^12^ The PRISMA 2020 statement was designed primarily to provide guidance for systematic reviews of studies that evaluate the effects of interventions. While the statement was intended for original, updated, or living systematic reviews, the developers noted that there might be additional reporting considerations that need to be addressed for LSRs.^10^ ^11^ Reporting considerations particularly relevant for LSRs include specifying changes between versions to the review questions (eg, any of the PICO elements—population, intervention, comparator, outcomes), methods (eg, eligibility criteria), results (eg, included studies, effect estimates), and conclusions.

Methodological studies have found variability in the reporting of some aspects of LSRs (eg, study flow diagrams^13^), and the absence of important information (eg, whether the LSR has been retired from a living mode^7^ ^14^). Another challenge is communicating with end users about differences between LSR versions; for example, differences in the list of eligible studies identified since the publication of the latest version.^15^ One of the likely reasons for these reporting issues is the absence of reporting guidance specific to the unique issues that arise in LSRs. To address these gaps, we developed an extension of the PRISMA 2020 statement for LSRs (PRISMA-LSR).

**Summary points**

The PRISMA-LSR checklist includes four new items addressing (L1) living mode parameters, (L2) changes to the methods, (L3) changes to the results, and (L4) authors and their roles for each version of the LSR. The checklist also includes new elements under some of the existing PRISMA 2020 items
After the latest version of an LSR has been published, the LSR status updates readers on whether the review is ongoing (and whether any new studies have been identified and are being incorporated) or whether the review has been retired
Templates are provided for four LSR tailored flow diagrams
The extension has been developed for full reports of LSRs; therefore, authors and journals need to define which items and elements of the checklist apply to a partial report of their LSR

## Development of PRISMA-LSR extension

We have reported the methods for developing the PRISMA-LSR extension.^16^ In brief, we first established a nine person executive committee (named authors of this article; see appendix 1 for details). We followed the EQUATOR Network’s guidance for developing health research reporting guidelines^17^ and registered the project.^18^ The Ethics Commission of the Faculty of Medicine of Cologne University provided ethical approval for the online survey (described below). We sought written informed consent from all participants for taking part in the online survey.

We conducted four background studies: a methodological survey of the reporting of flow diagrams in published LSRs^13^; a concept paper on methodological challenges with covid-19 reviews that were described as LSRs^19^; a methodological survey of published LSRs^14^; and a scoping review of the methodological literature on LSRs.^20^ Because of time constraints, we did not complete the planned interview study to explore authors’ views and experiences with conducting, reporting, and publishing LSRs.^16^ Early in the process, the executive committee discussed and agreed on the types of information that are specific to the reporting of LSRs (box 1).^21^ The discussion was informed by the findings of the background studies and members’ experience undertaking LSRs.

Box 1 **Types of information specific to the reporting of living systematic reviews (LSRs)^21^**

- Justification for adopting the living mode.
- LSR specific methods, referred to as living mode parameters; that is, the preset frequencies of conducting specific steps of the LSR or any triggers for conducting those steps sooner than planned.
- Changes between LSR versions, including changes to the general characteristics of the LSR (eg, authors’ declarations of interests, funding for the LSR), methods, findings, Preferred Reporting Items for Systematic reviews and Meta-Analyses (PRISMA) flow diagram, conclusions, and editorial process.
- LSR updating status (refer to glossary of terms; box 2).

The executive committee developed a first draft of the PRISMA-LSR extension based on the results of the background studies.^13^ ^14^ ^19^ ^20^ The draft followed the structure of the expanded PRISMA 2020 checklist, which includes 27 items, with detailed reporting recommendations for each item (called elements).^10^ ^11^ We did not alter the PRISMA 2020 items, but instead proposed elements specific to LSRs for most items. The executive committee nominated people representing different stakeholder groups (eg, systematic reviewers, guideline developers, editors, publishers) to form an international expert panel (PRISMA-LSR Group; see appendix 1). Of 76 people identified, 67 (88%) accepted our invitation to participate. Participation involved attending one of four initial online meetings (meeting replicated in different time zones) during March-April 2022 in which the project was introduced, and following this, completing an online survey to provide their views on whether the proposed elements in the draft PRISMA-LSR extension should be introduced without changes, introduced with changes, or omitted (appendix 2). Panel members were also able to provide comments, and to suggest new elements. The executive committee then met to discuss the quantitative and qualitative findings from the survey to decide whether the proposed elements should be kept the same, reworded or omitted, and whether new elements should be added. The committee used a 66% agreement by panel members on a specific element as a guide to decide on its inclusion. The executive committee also decided on elements that should be presented under new items (*v* elements that should be presented under the existing PRISMA 2020 items). This resulted in a second draft of the PRISMA-LSR extension. The expert panel provided feedback on the second draft during a series of seven online meetings (meeting replicated in different time zones) during October-November 2022 and by email. Subsequently, the executive committee considered the feedback and finalised the extension.

## Scope of PRISMA-LSR

Box 2 presents a glossary of terms used in the PRISMA-LSR statement. The PRISMA-LSR extension was designed primarily for LSRs that evaluate the effects of health interventions, irrespective of the design of the included studies or approach to synthesis, such as pairwise and network meta-analysis.^22^ However, the reporting guidance is also applicable to LSRs with objectives other than evaluating effects of health interventions (eg, those evaluating diagnostic test accuracy,^23^ prognostic factors, or prediction models). Furthermore, most of the guidance is applicable to standard updates of any type of systematic review. The PRISMA-LSR extension primarily provides reporting guidance for completed LSRs. However, authors preparing a protocol for an LSR should, in addition to adhering to the PRISMA for Protocols (PRISMA-P) 2015 statement^24^ (currently being updated), address specific PRISMA-LSR items. These are PRISMA-LSR items 1 (ie, title item), 3-15, and L1 (ie, all introduction and methods items except item L2, which addresses changes to the methods). The extension has been developed for full reports of LSRs. Authors and journals would need to define which items and elements of the checklist would apply to a partial report of their LSR, while referencing previous reports for information related to the remaining items and elements.

Box 2 **Glossary of terms**

- Living systematic review (LSR): a systematic review that is continually updated, incorporating relevant new evidence as it becomes available.^5^ This ideally leads to the regular publication of iterative versions over time (eg, weekly, monthly, quarterly, or as needed). Of note, the first LSR version is sometimes referred to as the base version.
- Living mode parameters: the preset frequencies of conducting specific steps of the LSR (eg, rerunning the search, updating the analysis, communicating new findings) or any triggers for conducting those steps.
- Full report of an LSR: a report that includes all the typical sections of a systematic review report, including introduction, methods, results, and discussion sections.
- Partial report of an LSR: a report that does not include all the typical sections of a systematic review report; it might include only a description of the changes between LSR versions and refers readers to a previous version of the review for other information; it might also include minimal information (eg, only an updated forest plot, a list of newly identified studies, a statement that no new studies have been found).
- Retirement from the living mode: decision to stop maintaining the review using the living mode.
- LSR status: information published after the publication of the latest LSR version to indicate whether that review is ongoing or has been retired (ie, living status of the review). If the LSR is ongoing, the status could also indicate whether any new studies have been identified, and whether they are being incorporated into the forthcoming version (ie, status of the evidence).

While developing the extension, we identified issues that are relevant to publishing aspects of LSRs as opposed to their reporting (eg, indicating whether the version being accessed by readers is the latest version of the LSR). We have restricted the checklist items to those pertinent to reporting, but we address publishing issues in the explanation and elaboration document (appendix 3). We did not address the use of artificial intelligence in PRISMA-LSR because it is not restricted to LSRs.^25^ ^26^

## How to use the PRISMA-LSR extension

The PRISMA-LSR extension includes the PRISMA-LSR checklist (table 1), the PRISMA-LSR flow diagram (figures S1-S4 presented in appendix 4), reporting recommendations for the LSR status (box 3), and an explanation and elaboration document (appendix 3). The PRISMA-LSR checklist and flow diagram are applicable for each version of the LSR, while the reporting recommendations for the LSR status can be used to update readers about the living status of the review and the status of the evidence in between published versions (box 2).

**Table 1:** Additional items (denoted with upper case L) and elements in PRISMA-LSR checklist compared with PRISMA 2020 checklist

Section and topic	PRISMA 2020 checklist items*	PRISMA-LSR checklist	
		Items	Elements
**Title**			
Title	1. Identify the report as a systematic review	—	• Identify the report as “living” in the title†
			• Provide the version number
**Abstract**			
Abstract	2. See PRISMA 2020 for abstracts checklist	—	• Indicate whether the LSR is being retired from the living mode after the publication of the current version, if applicable
**Introduction**			
Rationale	3. Describe the rationale for the review in the context of existing knowledge	—	• Justify the use of the living mode
		—	• Cite the preceding version of the LSR, if applicable
Objectives	4. Provide an explicit statement of the objective(s) or question(s) the review addresses	—	• Describe and justify any changes since the preceding version to the review's objective(s) or question(s)
**Methods**			
Living mode parameters	—	L1. Specify the living mode parameters	• Specify the planned schedule of the search for each source (eg, at a prespecified interval, following predefined triggers)
			• Specify the planned schedules for the remaining steps of the systematic review (eg, at a prespecified interval, following predefined triggers), if applicable. The remaining steps include screening, data collection, risk of bias assessment, analysis, certainty of evidence assessment, publication
			• Specify the plan for retirement from the living mode (eg, based on a prespecified timeline, following predefined triggers), if there is one. If there is no such plan, indicate so
Eligibility criteria	5. Specify the inclusion and exclusion criteria for the review and how studies were grouped for the syntheses	—	—
Information sources	6. Specify all databases, registers, websites, organisations, reference lists, and other sources searched or consulted to identify studies. Specify the date when each source was last searched or consulted	—	—
Search strategy	7. Present the full search strategies for all databases, registers and websites, including any filters and limits used	—	—
Selection process	8. Specify the methods used to decide whether a study met the inclusion criteria of the review, including how many reviewers screened each record and each report retrieved, whether they worked independently, and if applicable, details of automation tools used in the process	—	—
Data collection process	9. Specify the methods used to collect data from reports, including how many reviewers collected data from each report, whether they worked independently, any processes for obtaining or confirming data from study investigators, and if applicable, details of automation tools used in the process	—	• Describe whether the review team updated or planned to update collected data for a previously included study when relevant
Data items	10a. List and define all outcomes for which data were sought. Specify whether all results that were compatible with each outcome domain in each study were sought (eg, for all measures, time points, analyses), and if not, the methods used to decide which results to collect	—	—
	10b. List and define all other variables for which data were sought (eg, participant and intervention characteristics, funding sources). Describe any assumptions made about any missing or unclear information	—	—
Study risk of bias assessment	11. Specify the methods used to assess risk of bias in the included studies, including details of the tool(s) used, how many reviewers assessed each study and whether they worked independently, and if applicable, details of automation tools used in the process	—	• Describe whether the review team updated or planned to update risk of bias information for a previously included study when relevant
Effect measures	12. Specify for each outcome the effect measure(s) (eg, risk ratio, mean difference) used in the synthesis or presentation of results	—	—
Synthesis methods	13a. Describe the processes used to decide which studies were eligible for each synthesis (eg, tabulating the study intervention characteristics and comparing against the planned groups for each synthesis—item 5)	—	—
	13b. Describe any methods required to prepare the data for presentation or synthesis, such as handling of missing summary statistics, or data conversions	—	—
	13c. Describe any methods used to tabulate or visually display results of individual studies and syntheses	—	—
	13d. Describe any methods used to synthesise results and provide a rationale for the choice(s). If meta-analysis was performed, describe the model(s), method(s) to identify the presence and extent of statistical heterogeneity, and software package(s) used	—	• Report any analytical methods applied specifically because of the living mode
	13e. Describe any methods used to explore possible causes of heterogeneity among study results (eg, subgroup analysis, meta-regression)	—	—
	13f. Describe any sensitivity analyses conducted to assess robustness of the synthesised results	—	—
Reporting bias assessment	14. Describe any methods used to assess risk of bias due to missing results in a synthesis (arising from reporting biases)	—	—
Certainty assessment	15. Describe any methods used to assess certainty (or confidence) in the body of evidence for an outcome	—	—
Changes to the methods	—	L2. Describe changes to the methods	• Describe and justify any changes since the preceding version to the methods (items L1, 5-15)
			• If there are no changes to the methods, indicate so
			• Indicate whether the changes to the methods were applied to previously included studies
**Results**			
Study selection	16a. Describe the results of the search and selection process, from the number of records identified in the search to the number of studies included in the review, ideally using a flow diagram	—	• Describe what triggered the current version, if applicable
			• Ideally, use a flow diagram to illustrate the results of the search and selection processes in the different versions of the review using one of the LSR tailored flow diagrams
	16b. Cite studies that might appear to meet the inclusion criteria, but which were excluded, and explain why they were excluded	—	—
Study characteristics	17. Cite each included study and present its characteristics	—	—
Risk of bias in studies	18. Present assessments of risk of bias for each included study	—	—
Results of individual studies	19. For all outcomes, present, for each study: (a) summary statistics for each group (if appropriate) and (b) an effect estimate and its precision (eg, confidence or credible interval), ideally using structured tables or plots	—	—
Results of syntheses	20a. For each synthesis, briefly summarise the characteristics and risk of bias among contributing studies	—	—
	20b. Present results of all statistical syntheses conducted. If meta-analysis was done, present for each the summary estimate and its precision (eg, confidence or credible interval) and measures of statistical heterogeneity. If comparing groups, describe the direction of the effect	—	• Report results of any analytical methods applied specifically because of the living mode
	20c. Present results of all investigations of possible causes of heterogeneity among study results	—	—
	20d. Present results of all sensitivity analyses conducted to assess the robustness of the synthesised results	—	—
Reporting biases	21. Present assessments of risk of bias due to missing results (arising from reporting biases) for each synthesis assessed	—	—
Certainty of evidence	22. Present assessments of certainty (or confidence) in the body of evidence for each outcome assessed	—	—
Changes to the results	—	L3. Describe changes to the results	• Indicate the studies that were included since the preceding version (related to PRISMA 2020 item 17)
			•Describe and justify the changes since the preceding version in the eligibility status of any study (ie, excluding a previously included study, including a previously excluded study; related to PRISMA 2020 item 16)
			• Describe any other consequential changes since the preceding version to the results
			• If there are no changes to the results, indicate so
**Discussion**			
Discussion	23a. Provide a general interpretation of the results in the context of other evidence	—	—
	23b. Discuss any limitations of the evidence included in the review	—	—
	23c. Discuss any limitations of the review processes used	—	• Discuss any limitations related to the living mode
	23d. Discuss implications of the results for practice, policy, and future research	—	• Describe any changes since the preceding version to the implications of the results for practice, policy, and future research
			• Describe and justify any planned changes to review methods in upcoming review versions
			• Indicate and justify whether the LSR is being retired from the living mode following the publication of the current version, if applicable
**Other information**			
Registration and protocol	24a. Provide registration information for the review, including register name and registration number, or state that the review was not registered	—	—
	24b. Indicate where the review protocol can be accessed, or state that a protocol was not prepared	—	—
	24c. Describe and explain any amendments to information provided at registration or in the protocol‡	—	—
Support	25. Describe sources of financial or non-financial support for the review, and the role of the funders or sponsors in the review	—	• Describe the sources of financial or non-financial support and the roles of funders or sponsors in each of the versions of the LSR
Competing interests	26. Declare any competing interests of review authors	—	• Describe the competing interests of review authors and how they were managed for each of the versions of the LSR
Availability of data, code and other materials	27. Report which of the following are publicly available and where they can be found: template data collection forms; data extracted from included studies; data used for all analyses; analytic code; any other materials used in the review		• Describe any changes since the preceding version to the accessibility of data, code and materials
Authors and their roles for each version of the LSR	—	L4. Provide the list of authors and their roles for each version of the LSR	—

PRISMA-LSR=Preferred Reporting Items for Systematic reviews and Meta-Analyses 2020 statement for Living Systematic Reviews.

* Elements of the PRISMA 2020 checklist are not listed here. However, they should also be reported if applicable.

† While PRISMA 2020 item 1 included an additional (ie, non-essential) element to report whether “the review is a continually updated (‘living’) systematic review,” in PRISMA-LSR, this is an essential element.

‡ Item 24c of the PRISMA 2020 checklist is replaced by item L2 of the PRISMA-LSR checklist.

Box 3 **Reporting recommendations for living systematic review (LSR) status**

- Indicate whether the LSR is ongoing or being retired.
- If the LSR is being retired, provide reason(s).
- If the LSR is ongoing, indicate plans for the forthcoming version (including expected date of publication).
- If the LSR is ongoing, provide a list of studies identified as eligible since the publication of the latest version, and whether they are being incorporated into the forthcoming version.
- Provide the date the status was last updated.
- Indicate the date of the last search if run after publication of the previous version.

Given an LSR is a standard systematic review with additional features (box 2), reporting of LSRs should adhere to PRISMA 2020, but with additional information provided to address the unique LSR features. Accordingly, we have developed this extension as an add-on to the PRISMA 2020 statement, which should be used in addition to the PRISMA 2020 statement, and other relevant PRISMA extensions. For example, in the case of an LSR that includes a network meta-analysis, the PRISMA-LSR extension and PRISMA-NMA extension^27^ should be used alongside the PRISMA 2020 statement.^10^ ^11^ While the add-on approach might introduce complexity to the reporting of systematic reviews, efforts are underway to develop a web application to facilitate reporting when several extensions are relevant for the particular review.^28^

## PRISMA-LSR checklist

Table 1 presents the PRISMA-LSR checklist items and elements (right side of the table), along with the PRISMA 2020 checklist items (left side of the table). We have added four new items and 29 elements specific to the reporting of LSRs. Table S1 (appendix 5) provides a more detailed version of table 1 that includes the expanded PRISMA 2020 checklist (ie, with items and elements). 

The new items (denoted with upper case L) address reporting recommendations pertaining to (L1) living mode parameters, (L2) changes to the methods, (L3) changes to the results, and (L4) authors and their roles for each version of the LSR.

New elements added to existing PRISMA 2020 items include the following: identification of the report as “living” in the title (item 1), versioning (items 1 and 3), justification for using the living mode (item 3), plans for retirement from the living mode (items 2 and 23d), updating collected data and risk of bias information for a previously included study (items 9 and 11), analytical methods specific to the living mode (items 13d and 20b), a description of what triggered the current version (item 16a), illustration of results of the search and selection processes in the different versions (item 16a), limitations related to the living mode (item 23c), changes since the preceding version to the implications of the results (item 23d), planned changes to review methods in upcoming versions (item 23d), and changes since the preceding version to sources of support (item 25), competing interests (item 26), and accessibility of data, code, and materials (item 27).

Reporting recommendations pertaining to changes to the methods and results have been consolidated under items L2 and L3, respectively. However, this checklist structure should not be seen as prescriptive for where this information is reported. Authors might choose to report changes within the relevant section of the report (eg, changes to the statistical methods could be reported under the synthesis methods items (items 13a-f) instead of under item L2). Furthermore, we do not prescribe how authors report the changes (eg, by text, tabulation, or figures).

## PRISMA-LSR flow diagram

An important aspect of reporting in systematic reviews is the provision of a flow diagram that depicts the results of the search and selection processes. Four LSR tailored flow diagrams have been proposed^13^: presenting the search results of the different versions separately (ie, first version and each update separately); presenting the search results for the different versions combined (ie, including first version and all update versions); presenting the search results for the first version separately and the results of all update versions combined; and presenting the results of the latest update version separately and the results of all previous versions (including the first version) combined.^13^ While the first approach provides the most granular information, it is also the most cumbersome approach, and so might not be suitable as the number of updates increases. In this circumstance, authors might opt for one of the other approaches. We have developed an R package (https://github.com/nealhaddaway/livingPRISMAflow) and web based ShinyApp (https://estech.shinyapps.io/livingprismaflow) to create PRISMA 2020 flow diagrams for each of the four LSR tailored flow diagram approaches described above.^29^ Word templates for these flow diagrams are also provided in appendix 4.

## Reporting recommendations for LSR status

After the latest version of an LSR has been published, the LSR status updates readers on whether the review is ongoing (and whether any new studies have been identified and are being incorporated) or has been retired (box 2). Box 3 provides the reporting recommendations for the LSR status, and is intended to be used after the latest publication of an LSR version (ie, between review reports). When the journal publishing the different versions of the LSR does not publish the LSR status, authors might publish it elsewhere (eg, on a web page). Examples for the LSR status are presented in the explanation and elaboration document (appendix 3).

## Explanation and elaboration

In addition to the PRISMA-LSR checklist, an explanation and elaboration document is provided which gives an explanation (rationale and any supporting literature) for including all the items and elements (appendix 3). Furthermore, for each item and element, we provide examples of complete reporting.

The following sections provide the explanation and elaboration for the four new items of the PRISMA-LSR checklist: (L1) living mode parameters, (L2) changes to the methods, (L3) changes to the results, and (L4) authors and their roles for each version of the LSR.

### PRISMA-LSR item L1: specify the living mode parameters

Living mode parameters can be placed in a box to make them more prominent. Box 2 gives a definition of the term “living mode parameters.”

#### PRISMA-LSR element (item L1)

Specify the planned schedule of the search for each source (eg, at a prespecified interval, following predefined triggers).

Explanation: 

LSRs are characterised by frequent updates to the literature search, which can be done following different schedules^30^ (eg, fixed interval schedule, following predefined triggers). Predefined triggers could include LSR authors becoming aware of the publication of an eligible study (eg, through communication with colleagues or a press release). Examples of schedules also include automatic alerts and monitoring study registers. A methodological survey of 76 LSRs found that 33 (43%) reported a fixed interval schedule for updating; the median and interquartile range of the planned period of update being 3 months and 1-4.5 months.^14^ Most covid-19 related LSRs considered by a concept paper addressing methodological challenges for LSRs ran their search weekly or monthly.^19^ The planned schedule of the search is important to report so that users can assess the adequacy of the search update approach and understand how frequently the review will be updated. The planned schedule could be based on a specific rationale or simply on convenience.

Example: 

“An automated search is run every day, with results deduplicated and imported into Research Electronic Data Capture.”^31^

#### PRISMA-LSR element (item L1)

Specify the planned schedules for the remaining steps of the systematic review (eg, at a prespecified interval, following predefined triggers), if applicable. The remaining steps include screening, data collection, risk of bias assessment, analysis, certainty of evidence assessment, and publication.

Explanation: 

The planned schedules for the remaining steps of the systematic review could be the same as those of the search, or different. For example, a scoping review of the methodological literature and guidance on how to conduct, report, publish, and appraise the quality of LSRs found that the frequency for data abstraction could be determined by the continuous search (trigger dependent); immediately after study identification; or once new evidence has been identified for inclusion. Similarly, for quality and risk of bias assessment, updating could be regular, at a defined time interval, or occur once new evidence has been identified for inclusion. For data synthesis, updating could occur immediately after new study inclusion; on a continuous basis; or once new evidence has been identified for inclusion. Only one paper identified by that scoping review reported on the frequency of certainty of evidence assessment, which was after regular updating.^20^ Details about the planned schedules for the different steps of the systematic review are important to report so that users can assess the adequacy of methods and understand how frequently the review will be updated. The planned schedule could be based on a specific rationale or simply on convenience. Authors might decide to prioritise certain outcomes or to update some comparisons more frequently than others. In that case, they could report on the different updating frequencies.

Example: 

“Our aim is to update the synthesis at least once every week. For this purpose, we will search for, screen and extract data every day. The updated synthesis will be reported online at least once every week. In addition, we will update this Cochrane Review at least once every six months, or as soon as the certainty of evidence (assessed with the GRADE [Grading of Recommendations Assessment, Development and Evaluation] methodology) changes. We will wait until the accumulating evidence changes one or more of the following aspects of the review, before incorporating it and re‐publishing the Cochrane Review:

- the findings of one or more critical outcomes;
- the credibility (eg, GRADE rating) of one or more critical outcomes;
- new settings, population, interventions, comparisons or outcomes studied; or
- new serious adverse events.”^32^

#### PRISMA-LSR element (item L1)

Specify the plan for retirement from the living mode (eg, based on a prespecified timeline, following predefined triggers), if there is one. If there is no such plan, indicate so.

Explanation: 

When applicable, specifying plans for retiring the LSR from the living mode is important so that users can assess the adequacy of the plan and understand when updates are not to be expected anymore. This element recommends reporting the general plan for retirement from the living mode (box 2), while the third PRISMA-LSR element of item 23d recommends reporting the reason for retiring the LSR in question. Murad and colleagues proposed a number of triggers that might lead to an LSR being retired. These include when the evidence becomes conclusive, which can be determined based on certainty of the evidence or statistical methods; when the topic becomes less relevant to stakeholders; when new studies are not expected to be published; when required resources become unavailable^33^; however, other triggers might also be possible.

Examples:

- “We plan to update our literature search … every 2 months through December 2021 …”^34^
- “Each year, we will consider the necessity for the review to be a living systematic review by assessing ongoing relevance of the question to decision‐makers and by determining whether uncertainty is ongoing in the evidence and whether further relevant research is likely.”^35^

### PRISMA-LSR item L2: Describe changes to the methods

This item replaces the third and fourth elements of item 10a, and item 24c of the PRISMA 2020 checklist.

#### PRISMA-LSR element (item L2)

Describe and justify any changes since the preceding version to the methods (items L1, 5-15).

Explanation: 

The methods of an LSR might change across versions. Reasons for change could relate to the LSR question (eg, evolving understanding of the condition being studied) or to the LSR processes (eg, emergence of new tools). Such changes are important to report and justify. It might be too cumbersome for readers, and burdensome for authors, to report both changes since the preceding version and (cumulative) changes since the publication of the protocol; therefore, the extension recommends the former. LSR authors could, in addition, report on changes since the protocol. If authors decide to consolidate the changes to the methods in a section of the LSR, they would indicate in the PRISMA-LSR checklist the page(s) where the consolidated section is included. If authors decide to report changes within the relevant section of the LSR report, they would indicate in the PRISMA-LSR checklist the page(s) where the relevant methods subsections are included.

Examples of changes to the methods that could arise (identified by the expert panel) include the following:

- Relating to PRISMA 2020 item 5: Authors should always rely on the best available evidence, which will likely evolve and change rapidly over time.^19^ Therefore, eligibility criteria (eg, in terms of study design, types of publication) might change over the course of the LSR. For example, for some covid-19 related LSRs, authors initially included non-randomised studies, but later excluded these studies and included clinical trials.^19^
- Relating to PRISMA 2020 item 6: Databases might be discontinued or no longer be accessible, and new information sources could be added. A challenge reported for reviews conducted during the covid-19 pandemic was the dynamic nature of electronic databases.^19^ For example, the Centers for Disease Control and Prevention covid-19 Research Articles Downloadable Database, an early and comprehensive source of preprint articles, was discontinued in mid-2020, but was later completely covered by the World Health Organization covid-19 Global literature on coronavirus disease database.^19^ Refer to PRISMA-S for reporting on the search process.^36^
- Relating to PRISMA 2020 item 7: As understanding of the condition being studied and the evidence evolves, relevant terms, keywords, or database filters might change. According to the 2019 Cochrane guidance for LSRs, the search strategies need to be updated.^30^ We have kept it flexible for the authors to choose where to report the changes to the search strategies (eg, in the main text, in the appendix). The PRISMA 2020 checklist requires the reporting of the search strategy; that is, the latest search strategy (possibly modified from the preceding version) will be reported. The PRISMA-LSR extension requires the reporting of any modifications and their justifications. Previous versions of the search will be included in previous versions of the LSR and the readers can refer to these. The second PRISMA-LSR element of item 3 would facilitate the access of readers to the earlier version of the search strategy. For the search strategy, consider reporting only on “important” changes. Examples include use of a new search filter and removing a search block to increase sensitivity. Refer to PRISMA-S for reporting on the search strategies.^36^
- Relating to PRISMA 2020 item 8: To enhance efficiency during the course of the LSR, authors might apply changes to their screening process. Changes to this item include changes to any aspect of the screening process, such as application of the machine learning/automation tool or the use of crowdsourcing.
- Relating to PRISMA 2020 item 9: To enhance efficiency during the course of the LSR, authors might apply changes to their data abstraction process. Changes to this item include changes to any aspect of the data abstraction process, such as application of the machine learning/automation tool or the use of crowdsourcing.
- Relating to PRISMA 2020 item 10: New outcomes might be added in subsequent versions of an LSR with emerging information about new outcomes (eg, covid vaccines and vaccine induced immune thrombotic thrombocytopenia). Changes in outcomes include the measurement of an outcome at a new follow-up time point, and modification of a core outcome set. Authors might cease to update the analyses for a specific outcome in the living mode.
- Relating to PRISMA 2020 item 11: As eligibility by study design might change during the course of the LSR, authors should report on changes in the risk of bias assessment tool(s) used. To enhance efficiency during the course of the LSR, authors might apply changes to their risk of bias assessment process. Changes to the latter include changes in application of the machine learning/automation tool or the use of crowdsourcing.
- Relating to PRISMA 2020 item 12: Effect measures might be added or changed (eg, relative risk to hazard ratio); for example, when new studies become available. Changes to this item also include a change in thresholds used to interpret the size of effect.
- Relating to PRISMA 2020 item 13: As the evidence evolves, authors might decide to plan for new analyses.
- Relating to PRISMA 2020 item 15: As the evidence evolves, authors might decide to change the list of comparisons and outcomes subject to certainty assessment.

Examples:

- Example relating to PRISMA-LSR item L1: “Since August 2021, we have run the searches monthly instead of weekly.”^37^
- Example relating to PRISMA 2020 item 5: “We amended eligibility criteria after the third version of the review (reference) in 2 ways. First, we excluded studies that only reported the proportion of presymptomatic SARS-CoV-2 because the settings and methods of these studies were very different and their results were too heterogeneous to summarise (reference) …”^38^
- Example relating to PRISMA 2020 item 6: “We stopped searching the Chinese databases on 20 February 2021 because they had not provided studies that meaningfully altered the evidence for any intervention.”^39^
- Example relating to PRISMA 2020 item 7: “At the beginning of 2021, new MeSH or EMTREE terms were inserted in Medline and Embase, so the whole search strategies were revised and new search terms like IGY‐110 or GIGA‐2050 or GC5131 or 5131A or INOSARS were added.”^37^
- Example relating to PRISMA 2020 item 8: “Due to the increased volume of published and preprint articles, we used artificial intelligence text analysis from 25 May 2020 and onwards to conduct an initial classification of documents, based on their title and abstract information, for relevant and irrelevant documents.”^40^
- Example relating to PRISMA 2020 item 9: “We had planned to extract data using a standardised data extraction form developed in Covidence. However, we could not adapt the standardised form to our needs. Therefore we generated a customised data extraction form in Microsoft Excel (Microsoft Corporation 2018).”^37^
- Example relating to PRISMA 2020 item 10: “We renamed the outcome ‘time to discharge from hospital’ to ‘Duration of hospitalisation, or time to discharge from hospital’ to clarify that we are interested in both, continuous and time‐to‐event data.”^37^
- Example relating to PRISMA 2020 item 11: “After the third version of the review (reference), we developed a new tool to assess the risk of bias because the study designs of included studies have changed.”^38^
- Example relating to PRISMA 2020 item 13: “We had added subgroup analyses for the following characteristics in this update of the review.
  o Duration since symptom onset
  o Level of antibody titre in donors
  o Level of antibody titre in recipients at baseline
  o SARS‐CoV‐2 variants
  o Considering the currently available evidence, we decided to add these subgroups, because their role in the effectiveness of convalescent plasma is currently being discussed and needs to be further investigated.”^37^
- Example relating to PRISMA 2020 item 14: “In case of missing data, we conducted an available‐case analysis” (under differences between fourth and current published review version).”^37^
- Example relating to PRISMA 2020 item 15: “At protocol stage, we had planned to assess the certainty in the evidence for our primary outcomes (all‐cause mortality at hospital discharge and time to death) only. However, as none of the included studies reported any deaths during their study periods, we decided to assess the certainty in the evidence also for prioritised secondary outcomes (clinical improvement, grades 3 and 4 adverse events, and serious adverse events) to increase the informative value on effectiveness and safety of convalescent plasma therapy.”^37^

#### PRISMA-LSR element (item L2)

If there are no changes to the methods, indicate so.

Explanation: 

Providing a general statement about the absence of any changes to the methods, or reporting that particular methods did not change, indicates to returning readers that they might not need to reread the particular methods section.

Example: 

“Tools to assess risk of bias and estimate certainty of evidence (COE) were unchanged.”^41^

#### PRISMA-LSR element (item L2)

Indicate whether the changes to the methods were applied to previously included studies.

Explanation: 

It is possible for changes to the methods of an LSR to be consequential on previously included studies. Therefore, authors should indicate whether the changes to the methods were applied to previously included studies (eg, repeating risk of bias assessment for a previously included study based on the use of a new risk of bias assessment tool). When changes were not applied to previously included studies, this should be reported.

Example: 

No examples found.

### PRISMA-LSR item L3: Describe changes to the results

In general, highlighting changes to the results is particularly helpful for returning readers. Examples of what can cause changes to the results are the inclusion of new studies, changes related to the already included studies (eg, retraction of a study, obtaining new information from study authors), or changes in the LSR methods (eg, use of a new risk of bias tool). When the reason for changes to the results is not obvious, it is important to describe the reason for the changes. It might be too cumbersome for readers, and burdensome for authors, to report both changes since the preceding version and (cumulative) changes since the publication of the protocol; therefore, the extension recommends the former. LSR authors could, in addition, report on changes since the protocol. If authors decide to consolidate the changes to the results in a section of the LSR, they would indicate in the PRISMA-LSR checklist the page(s) where the consolidated section is included. If authors decide to report changes within the relevant section of the LSR report, they would indicate in the PRISMA-LSR checklist the page(s) where the relevant results subsections are included.

#### PRISMA-LSR element (item L3)

Indicate the studies that were included since the preceding version (related to PRISMA 2020 item 17).

Explanation: 

Indicating the studies that were included since the preceding version should be reported. Highlighting these studies might decrease the burden for returning readers. This element applies to both studies and reports of studies.

Example: 

“We included one study for outpatients and one study for inpatients in our qualitative synthesis. One study was in the original review (reference), and one study from this update (reference).”^42^

#### PRISMA-LSR element (item L3)

Describe and justify the changes since the preceding version in the eligibility status of any study (ie, excluding a previously included study, including a previously excluded study; related to PRISMA 2020 item 16).

Explanation: 

It is important for readers to be aware of any changes to the eligibility status of studies and the reasons for the changes. The justification could be related to a change in LSR methods (eg, eligibility), or to obtaining new information from newly available reports (including withdrawal or retraction of a study, expression of concerns) or from personal contact with authors. Newly available reports refer to peer reviewed publications, preprints, grey literature, a web page, etc. The status of studies labelled as “ongoing” and “awaiting classification” in the preceding LSR version should be updated, if applicable.

Example: 

“Since the first iteration, one trial addressing ivermectin and showing large positive effects was retracted. The living nature of our systematic review and network meta-analysis enables the exclusion of retracted data from this second iteration and between subsequent iterations if needed.”^43^

#### PRISMA-LSR element (item L3)

Describe any other consequential changes since the preceding version to the results.

Explanation: 

Reporting consequential changes to the results is recommended. These changes include those that are likely to impact the interpretation of the effect size (eg, small to moderate effect size), and the certainty assessment. LSR authors could, in addition, report on any changes to the results.

Examples of changes to the results that could arise (identified by the expert panel) include the following:

- Relating to PRISMA 2020 item 17: Characteristics of a previously included study might be subject to change based on newly available reports, including errata. Newly available reports refer to peer reviewed publications, preprints, grey literature, a web page, etc. A specific example is platform trials (ie, “trials that study multiple targeted therapies in the context of a single disease in a perpetual manner, with therapies allowed to enter or leave the platform on the basis of a decision algorithm”^44^), which might include new interventions between review updates, or might conduct a nested study within a different original study design. In these cases, authors would report on a change in study design or study intervention. Updating the primary references of already included studies is another example of a change worth documenting.
- Relating to PRISMA 2020 items 18 and 21: Consequential changes could be related to a change in the risk of bias tool used by the authors to obtain new information from newly available reports (eg, checking whether data previously abstracted from a preprint have changed in the peer reviewed paper, abstracting new data) or from personal contact with authors. Newly available reports refer to peer reviewed publications, preprints, grey literature, a web page, etc.
- Relating to PRISMA 2020 item 20b: Indicate which outcomes have results of statistical syntheses available for the first time. This includes a measurement of an outcome at a new follow-up time point. Indicate syntheses that have been removed since the preceding version.
- Relating to PRISMA 2020 item 20c: Indicate outcomes for which investigations of possible causes of heterogeneity have results available for the first time. Indicate investigations of possible causes of heterogeneity that have been removed since the preceding version.
- Relating to PRISMA 2020 item 20d: Indicate outcomes for which results of sensitivity analyses are available for the first time. Indicate sensitivity analyses that have been removed since the preceding version.
- Relating to PRISMA 2020 item 22: Change in certainty of evidence should be reported when applicable for each outcome. Refer to detailed GRADE guidance for explanatory footnotes to support changes in the GRADE certainty in the evidence judgments.^45^

Examples:

- Example relating to PRISMA 2020 item 17: “Twelve preprints were subsequently published after peer review. The supplementary data present the differences between study preprint and peer reviewed publications.”^46^
- Example relating to PRISMA 2020 item 18: “Published reports of 3 studies previously available as preprints became available (references), enabling more thorough assessment for risk of bias. The risk of bias is now determined to be serious for Yu and colleagues' study, remains high for Tang and colleagues' study, and changed from moderate to serious for Mahévas and colleagues' study.”^47^
- Example relating to PRISMA 2020 item 20b: “Figure 16 displays the pooled sensitivity and specificity estimates with 95% confidence intervals from all four versions of this review (ie, Salameh 2020a published in September 2020, Islam 2020 published in November 2020, Islam 2021 published in March 2021, and this current version). The sensitivity estimates of chest CT appear to be similar across McInnes 2020, Islam 2020, Islam 2021 and this current version, while the specificity estimates of chest CT appear to increase from Salameh 2020a to Islam 2021, and then remain similar between version 3 and the current version.”^40^
- Example relating to PRISMA 2020 item 20c: “The correction of analysis 17.2 changed the conclusion for the subgroup analysis age of participants, as the test for subgroup differences was not significant anymore.”^37^
- Example relating to PRISMA 2020 item 22: “The newly included randomized controlled trials strengthen previous findings on the benefit of remdesivir on the proportion of patients receiving ventilation or extracorporeal membrane oxygenation at follow-up but decreases the strength of previous findings on the reduction of serious adverse events with remdesivir.”^41^

#### PRISMA-LSR element (item L3)

If there are no changes to the results, indicate so.

Explanation: 

Providing a general statement about the absence of any changes to the results, or indicating that particular results did not change, indicates to returning readers that they might not need to reread the particular results section.

Example: 

“Overall, the addition of the new studies and the retraction of 1 prior study does not change the findings or certainty of evidence ratings we reported in the original review.”^48^

#### PRISMA-LSR item L4: Provide the list of authors and their roles for each version of the LSR

Explanation: 

It is possible that the relative contributions of authors will vary across versions of an LSR, and that the author team will change across versions.^30^ Indeed, in a mixed methods evaluation with participants involved in Cochrane and non-Cochrane LSRs, participants discussed authorship issues as a complexity in the production of LSRs. Participants mentioned that the first versions of the reviews have a large authorship team while the subsequent versions with smaller changes required a much smaller team. The opportunity for contribution is further restricted by the speed of the updates. This led the author teams to question when people should come off the author list, and to request more guidance around this issue.^49^ This element highlights that LSRs should list all authors and their roles at any time, acknowledging authors of previous versions who may not be authors of the current version. This element could be reported in the format of a table, with information for different versions displayed in different rows. Authors could have their competing interests listed in the same table. Refer to the CrediT (Contributor Roles Taxonomy) system for reporting on author roles.^50^

Example: 

“The list of authors has changed between the protocol and the first review version, and has also changed with each update version. Changes to the author list since the protocol to the current review version are outlined below.”^40^

## Discussion

LSRs have been proposed as an innovative approach in the evidence synthesis field to enable frequent and rapid incorporation of evidence. The publication rate of LSRs has increased considerably in recent years.^4^ The PRISMA-LSR extension aims to facilitate transparent, complete, and accurate reporting of LSRs.

There are several strengths of our approach to developing an extension of the PRISMA 2020 statement for living systematic reviews (PRISMA-LSR). We followed the EQUATOR Network’s guidance. The membership of the executive committee as well as the expert panel represented a diversity of backgrounds and expertise. One limitation is that we were not able to recruit consumers to be part of this study; we plan to do so in future updates. Also, owing to resource limitations, we had to conduct the expert panel meetings online (as opposed to in person), and because of differences in time zones, we could not identify a common time for all the panellists to join a single session.

The extension benefits from the concept of using the add-on approach, which would facilitate the reporting of a systematic review for which several PRISMA extensions apply (eg, a rapid LSR with network meta-analysis), minimising the time and efforts of reviewers. It would be ideal to have a PRISMA web application where the systematic reviewer can check off features of the review (eg, rapid, living, and network meta-analysis) to obtain an integrated reporting checklist.^28^ Nonetheless, the efficiency of such integrated checklists and their effectiveness at improving reporting would need to be tested.

Also, we have allowed for flexibility in how authors implement the reporting guidance, in terms of what to report in a partial report, how to report the changes (eg, tabular versus changes tracked), whether to consolidate the changes or report them within the relevant section of the report, the version of the PRISMA-LSR flow diagram to use, and accounting for new LSR conduct methods and publication models and technology that are likely to emerge.

We acknowledge that LSRs face major challenges beyond adequate reporting, including the vast workload needed to maintain an LSR,^7^ ^19^ as well as the publication platforms not being fit for purpose. Implementation of this extension would be facilitated by dynamic publication platforms. Such platforms could allow for the automated and easy transfer of text when authors are writing a newer version of the LSR. Furthermore, they could ensure easy access to the most recent version of the LSR, earlier versions, and track changes between versions. This approach could reduce the workload for LSR authors and the editorial team, and facilitate accessibility to the needed information for new and returning readers. Although such publication platforms are not yet available, some of their features are currently being used. For example, Cochrane's “what's new” section and F1000Research's “update box” enable the reporting of changes in a reader friendly manner.^51^ ^52^ In the meantime, it is key that LSR authors report important information in the best way possible, which might involve more than one publication venue (eg, reporting versions of the review on a study webpage).

More research is needed to inform best reporting practices for LSRs and how they could facilitate the use of their findings to impact practice. For example, it would be worthwhile to conduct focus groups or interviews with authors, peer reviewers, publishers, and end users to determine the most acceptable formats to relay changes to the readers, what should be included in a partial report of an LSR, or which PRISMA flow diagram for LSRs is most preferred. Some of this research could be done as “study within a review” (SWAR).^53^ We plan to monitor for emerging evidence relevant to this reporting guidance for a future update. In addition, many aspects of LSR publishing that were identified through our work on this extension (eg, inclusion of a stable link to access the LSR protocol, linkage to the latest version of the LSR) should be explored further.

We have posted this extension on the PRISMA statement website (http://www.prisma-statement.org). We encourage readers and users to submit any comments or feedback by contacting the corresponding author. Finally, we welcome efforts to translate the PRISMA-LSR extension to other languages. We encourage journal editors and publishers to endorse the extension in addition to the PRISMA 2020 statement and include it in journals’ “Instructions to authors.”

### Conclusion

We hope that implementing the PRISMA-LSR reporting guidance will lead to more transparent, complete, and accurate accounts of LSRs. The extension is expected to benefit authors, editors, and peer reviewers of LSRs, and different users of LSRs, including guideline developers, policy makers, healthcare providers, patients, and other stakeholders by providing the necessary synthesised evidence to underpin healthcare decisions.

## Web extra

Extra material supplied by authors

Web appendix 1: PRISMA-LSR contributors

Web appendix 2: Expert panel survey

Web appendix 3: Explanation and elaboration

Web appendix 4: Figures S1-S4

Web appendix 5: Table S1

## Notes

Author contributors (PRISMA-LSR Group): Tony Aburrow, Lisa Askie, Vivienne C Bachelet, Asma Ben Brahem, Lex Bouter, Romina Brignardello-Petersen, Jacob Burns, Anna Chaimani, Christine Chang, Stephanie Chang, Mike Clarke, Kerry Dwan, Markus Follmann, Christian Gluud, Gordon Guyatt, Lotty Hooft, Anna Jacobs, Svenja Krebs, Tamara Kredo, Toby Lasserson, Helen Macdonald, Iain Marshall, Steve McDonald, Matthew DF McInnes, Joerg Meerpohl, Maria-Inti Metzendorf, David Moher, Lorenzo Moja, Karel GM Moons, Mohammad Hassan Murad, Reem Mustafa, Stefano Negrini, Robby Nieuwlaat, Adriani Nikolakopoulou, Monika Nothacker, Amir Qaseem, Gabriel Rada, Melissa L Rethlefsen, Ludovic Reveiz, Maria Ximena Rojas-Reyes, Jean-Paul Salameh, Georgia Salanti, Ian J Saldanha, Jochen Schmitt, Holger J Schünemann, Richard Sever, Mark Simmonds, Bhagteshwar Singh, Anneliese Synnot, Britta Tendal Jeppesen, James Thomas, David Tovey, Andrea Tricco, Tari Turner, Per Olav Vandvik, Helen Wakeford, Stephanie Weibel, Vivian Welch, Wojtek Wiercioch.

Non-author contributors (PRISMA-LSR Group): Sabina Alam, Isabelle Boutron, Tammy Clifford, Julian Elliott, Robin Featherstone, Elizabeth Loder, Rose O'Dea, Ashrita Saran.

Contributors: EAA, CI, VP, LAK, JMB, JEM, MJP, and NS conceptualised and designed the development of the extension. CI and JK contributed to project administration. EAA, JK, CI, VP, JMB, JEM, MJP, and NS developed the first draft of the extension. The PRISMA-LSR Group provided feedback on the extension. EAA, JK, CI, VP, JMB, JEM, MJP, and NS analysed the data. EAA and JK wrote the original draft of the manuscript. CI, VP, LAK, JMB, JEM, MJP, NS, and the PRISMA-LSR Group critically revised the manuscript and approved the final version. EAA and JK contributed equally. EAA is the guarantor of the work. The corresponding author attests that all listed authors meet authorship criteria and that no others meeting the criteria have been omitted.

Dissemination to participants and related patient and public communities: We plan to disseminate the research widely through presentations at conferences and through social media to interest-holders who generate or use evidence, including to consumers in evidence synthesis organisations.

Provenance and peer review: Not commissioned; externally peer reviewed.
