# Supplementary material for: Extension of the PRISMA 2020 statement for living systematic reviews (PRISMA-LSR): checklist and explanation
Source: BMJ. 2024 Nov 19;387:e079183. doi: 10.1136/bmj-2024-079183 (PMC12036629; doi:10.1136/bmj-2024-079183)
Supplement: Supplementary file 1 — Web appendix 1: PRISMA-LSR contributors [file akle079183.ww1.pdf]

Appendix 1: PRISMA-LSR contributors

PRISMA-LSR contributors

1. Members of the executive committee

- Elie A. Akl
- James M. Barker
- Claire Iannizzi
- Lara Kahale
- Joanne Khabsa
- Joanne E. McKenzie
- Matthew J. Page
- Vanessa Piechotta
- Nicole Skoetz

2. Members of the expert panel

**Name**	**Affiliation**	**Stakeholder category**
Tony Aburrow	Cochrane, London, UK	Publisher
Sabina Alam	Publishing Ethics and Integrity, Taylor and Francis Group Journals, Oxfordshire, United Kingdom	Publisher
Lisa Askie	World Health Organization (WHO), 20 Avenue Appia,1211 Geneva, Switzerland	Systematic reviewer<br>Methodologist<br>Funder
Vivienne C. Bachelet	Escuela de Medicina, Facultad de Ciencias Médicas, Universidad de Santiago de Chile (USACH), Santiago, Chile	Editor
Asma Ben Brahem	Guidelines and Care Pathways, INEAS (National Authority for Assessment and	Producer of health technology assessments

Name	Affiliation	Stakeholder category
	Accreditation in Healthcare), Tunis, Tunisia	
Lex Bouter	Amsterdam University Medical Center, Amsterdam, the Netherlands	Methodologist<br>Systematic reviewer
Isabelle Boutron	Université Paris Cité and Université Sorbonne Paris Nord, Inserm, INRAe, Centre for Research in Epidemiology and Statistics (CRESS), F-75004 Paris, France	Systematic reviewer
Romina Brignardello-Petersen	Department of Health Research Methods, Evidence, and Impact McMaster University, Hamilton, ON, Canada	Systematic reviewer
Jacob Burns	Institute for Medical Information Processing, Biometry and Epidemiology, LMU Munich, Munich, Germany	Systematic reviewer
Anna Chaimani	Université Paris Cité and Université Sorbonne Paris Nord, Inserm, INRAE, Center for Research in Epidemiology and Statistics (CRESS), F-75004 Paris, France<br><br>Cochrane France, Paris, France	Statistician
Christine Chang	Agency for Healthcare Research and Quality, Rockville, MD, USA	Funder
Stephanie Chang	Annals of Internal Medicine, Philadelphia, PA, USA	Editor
Mike Clarke	Northern Ireland Methodology Hub, Queen's University Belfast, Belfast, United Kingdom	Systematic reviewer (public health and policy)
Tammy Clifford	School of Epidemiology and Public Health, University of Ottawa, Ottawa, ON, Canada<br><br>Canadian Institute of Health Research, Government of Canada, Ottawa, ON, Canada	Funder

Name	Affiliation	Stakeholder category
Kerry Dwan	Liverpool School of Tropical Medicine, Liverpool, UK	Systematic reviewer
Julian Elliott	Cochrane Australia, School of Public Health and Preventive Medicine, Monash University, Melbourne, Victoria, Australia	Software developer
Robin Featherstone	Evidence Production and Methods Directorate, Cochrane, London, UK<br><br>Research Information Services, CADTH (Canadian Agency for Drugs and Technologies in Health), Ottawa, Canada	Information specialist
Markus Follmann	Office of the German Guideline Program in Oncology (GGPO), c/o German Cancer Society, Berlin, Germany	Guideline methodologist
Christian Gluud	Copenhagen Trial Unit, Centre for Clinical Intervention Research, The Capital Region, Copenhagen University Hospital – Rigshospitalet, Blegdamsvej 9, 2100, Copenhagen, Denmark<br><br>Department of Regional Health Research, The Faculty of Health Sciences, University of Southern Denmark, Odense, Denmark	Systematic reviewer
Gordon Guyatt	Department of Health Research Methods, Evidence, and Impact, McMaster University, Hamilton, ON, Canada	Systematic reviewer<br>Guideline methodologist
Lotty Hooft	Julius Center for Health Sciences and Primary Care, University Medical Center Utrecht, Utrecht University, Utrecht, The Netherlands<br><br>Cochrane Netherlands, University Medical Center Utrecht, Utrecht University, Utrecht, The Netherlands	Author of PRISMA extension(s)<br>Systematic reviewer<br>Guideline methodologist
Anna Jacobs	DLR Projektträger, Health Division, Bonn, Germany	Project management agency (managing funding initiatives on behalf of the German

Name	Affiliation	Stakeholder category
		Federal Ministry of Education and Research)
Svenja Krebs	DLR Projektträger, Health Division, Bonn, Germany	Project management agency (managing funding initiatives on behalf of the German Federal Ministry of Education and Research)
Tamara Kredo	Health Systems Research Unit, South African Medical Research Council	Systematic reviewer<br>Guideline methodologist
Toby Lasserson	Cochrane, London, UK	Editor<br>Systematic reviewer
Elizabeth Loder	Department of Neurology, Brigham and Women's Hospital, Boston, Massachusetts<br><br>Harvard Medical School, Boston, Massachusetts<br><br>BMJ, London, United Kingdom	Editor
Helen Macdonald	BMJ, London, UK	Editor
Iain Marshall	School of Life Course and Population Sciences, King's College London, London, UK	Software developer
Steve McDonald	School of Public Health and Preventive Medicine, Monash University	Information specialist
Matthew DF McInnes	University of Ottawa Departments of Radiology and Epidemiology; The Ottawa Hospital Research Institute	Author of PRISMA extension(s)
Joerg Meerpohl	Institute for Evidence in Medicine, Medical Center & Medical Faculty, University of Freiburg, Freiburg, Germany<br><br>Cochrane Germany, Cochrane Germany Foundation, Freiburg, Germany	Systematic Reviewer<br>Guideline methodologist
Maria-Inti Metzendorf	Institute of General Practice, Medical Faculty of the Heinrich-Heine University, Heinrich Heine University	Information specialist<br>Systematic reviewer

Name	Affiliation	Stakeholder category
	Düsseldorf, Düsseldorf, Germany	
David Moher	Centre for Journalology, Clinical Epidemiology Programme, Ottawa Hospital Research Institute, Ottawa, Ontario, Canada<br><br>School of Epidemiology and Public Health, University of Ottawa, Ottawa, Ontario, Canada	Author of the PRISMA statement and PRISMA extension(s)
Lorenzo Moja	Health Products Policy and Standards, World Health Organization, Geneva, Switzerland	Systematic reviewer (public health and policy)
Karel G. M. Moons	Julius Center for Health Sciences and Primary Care, UMC Utrecht, Utrecht University, Utrecht, The Netherlands	Systematic reviewer<br>Author of PRISMA extension(s)<br>Editor
Mohammad Hassan Murad	Evidence-based Practice Center. Mayo Clinic, Rochester, MN, USA	Systematic reviewer<br>Guideline methodologist
Reem Mustafa	Department of Internal Medicine, University of Kansas Medical Center, Kansas City, KS, USA<br><br>Department of Health Research methods, Evidence and Impact, McMaster University, Hamilton, ON< Canada	Systematic reviewer<br>Guideline methodologist
Stefano Negrini	Department of Biomedical Surgical and Dental Sciences, University "La Statale", Milan, Italy<br><br>IRCCS Istituto Ortopedico Galeazzi, Milan, Italy	Systematic reviewer<br>Editor
Robby Nieuwlaat	Michael G. DeGroote Cochrane Canada and MacGRADE Centres, McMaster University, Hamilton, ON, Canada<br><br>Department of Health Research Methods, Evidence, and Impact, McMaster University, Hamilton, ON, Canada	Systematic reviewer
Adriani Nikolakopoulou	Institute of Medical Biometry and Statistics, Faculty of Medicine and	Statistician

Name	Affiliation	Stakeholder category
	Medical Center - University of Freiburg, Freiburg, Germany	
Monika Nothacker	Association of the Scientific Medical Societies in Germany, Institute for Medical Knowledge Management	Guideline methodologist
Rose O'Dea	The School of Agriculture, Food and Ecosystem Sciences, The University of Melbourne, Melbourne, Victoria, Australia	Author of PRISMA extension(s)
Amir Qaseem	American College of Physicians, Philadelphia, Pennsylvania	Guideline methodologist
Gabriel Rada	Epistemonikos Foundation, Santiago, Chile<br><br>UC Evidence Centre, Pontificia Universidad Católica de Chile, Santiago, Chile	Systematic reviewer
Melissa L. Rethlefsen	Health Sciences Library & Informatics Center, University of New Mexico	Information specialist<br>Author of PRISMA extension(s)
Ludovic Reveiz	Science and Knowledge Unit, Department of Evidence and Intelligence for Action in Health, WHO Regional Office for the Americas/Pan American Health Organization, Washington, DC, USA	Systematic reviewer (public health and policy)
Maria Ximena Rojas-Reyes	Institute of Research (IR SANT PAU), Barcelona, Catalunya, Spain	Systematic reviewer<br>Guideline methodologist
Jean-Paul Salameh	Faculty of Health Sciences, Queen's University, Kingston, Ontario, Canada	Author of PRISMA extension(s)
Georgia Salanti	Institute of Social and Preventive Medicine (ISPM), University of Bern, Bern, Switzerland	Systematic reviewer<br>Statistician
Ian J. Saldanha	Johns Hopkins Bloomberg School of Public Health, Baltimore, USA	Systematic reviewer<br>Guideline methodologist<br>Software developer<br>Editor

Name	Affiliation	Stakeholder category
Ashrita Saran	Global Development Network, New Delhi, India	Systematic reviewer
Jochen Schmitt	Center for Evidence-Based Healthcare (ZEGV), University Hospital Carl Gustav Carus and Carl Gustav Carus Faculty of Medicine, TU Dresden, Dresden, Germany	Systematic reviewer
Holger J. Schünemann	Department of Health Research Methods, Evidence and Impact, McMaster University, Hamilton, Ontario, Canada<br><br>Michael G. DeGroote Cochrane Canada, MacGRADE Centres, McMaster University, Hamilton, Ontario, Canada<br><br>Department of Biomedical Sciences, Humanitas University, Milan, Italy	Systematic reviewer<br>Guideline methodologist
Richard Sever	Cold Spring Harbor Laboratory, New York, USA	Editor<br>Preprint server Co-Founder
Mark Simmonds	Centre for Reviews and Dissemination, University of York, York, North Yorkshire, UK	Statistician
Bhagteshwar Singh	Tropical & Infectious Diseases Unit, Royal Liverpool Hospital, Liverpool, UK<br><br>Institute of Infection, Veterinary & Ecological Sciences, University of Liverpool, Liverpool, UK	Guideline methodologist<br>Systematic reviewer
Anneliese Synnot	Australian Living Evidence Collaboration, School of Public Health and Preventive Medicine, Monash University, Melbourne, Australia<br><br>Centre for Health Communication and Participation, Department of Public Health, La Trobe University, Melbourne, Australia	Systematic reviewer<br>Guideline methodologist
Britta Tendal Jeppesen	Future Evidence Foundation<br><br>School of Public Health and Preventive	Systematic reviewer<br>Guideline methodologist

Name	Affiliation	Stakeholder category
	Medicine, Monash University, Melbourne, VIC, Australia	
James Thomas	EPPI-Centre, UCL Social Research Institute, University College London, London, UK	Systematic reviewer<br>Software developer
David Tovey	Cochrane France, Paris, France	Editor
Andrea Tricco	Unity Health Toronto, University of Toronto	Author of the PRISMA statement and PRISMA extension(s)<br>Editor
Tari Turner	School of Public Health and Preventive Medicine, Monash University	Guideline methodologist
Per Olav Vandvik	Department of Medicine, Lovisenberg Diaconal Hospital, Oslo, Norway (P.O.V.)	Guideline methodologist
Helen Wakeford	Department of Editorial and Methods, Cochrane Central Executive, London, UK	Systematic reviewer
Stephanie Weibel	University Hospital Wuerzburg, Department of Anaesthesiology, Intensive Care, Emergency and Pain Medicine	Systematic reviewer
Vivian Welch	Bruyere Research Institute, Ottawa, Canada	Systematic reviewer
Wojtek Wiercioch	Department of Health Research Methods, Evidence, and Impact, Michael G. DeGroote Cochrane Canada and GRADE Centres, McMaster University, Hamilton, ON, Canada;	Systematic reviewer<br>Guideline methodologist
