# Supplementary material for: Extension of the PRISMA 2020 statement for living systematic reviews (PRISMA-LSR): checklist and explanation
Source: BMJ. 2024 Nov 19;387:e079183. doi: 10.1136/bmj-2024-079183 (PMC12036629; doi:10.1136/bmj-2024-079183)
Supplement: Supplementary file 3 — Web appendix 3: Explanation and elaboration [file akle079183.ww3.pdf]

Appendix 3: Extension of the PRISMA 2020 statement for Living Systematic Reviews (PRISMA-LSR): explanation and elaboration (E&E)

1. Introduction

This document represents the Explanation and Elaboration (E&E) for the Preferred Reporting Items for Systematic reviews and Meta-analyses (PRISMA) extension for living systematic reviews (PRISMA-LSR). The format of this document is like that used in other explanation and elaboration documents related to the PRISMA reporting guidelines.[4-8]

We encourage readers to refer to the PRISMA-LSR document for how to use the extension along with the PRISMA 2020 statement. Briefly, this extension is conceived as a module that can be used in addition to the PRISMA 2020 statement to address the living mode aspects of a systematic review. In other words, the PRISMA-LSR extension provides items and reporting recommendations (ie, elements) specific to the living mode, to be used in addition to those listed in the PRISMA 2020 statement. While extension items are independent of the PRISMA 2020 items, the extension elements might be sitting under the extension (new) items or under the PRISMA 2020 (existing) items.

For each of the elements, and for better context, we include the item under which they are listed, and indicate whether it is a new (from the extension) or an existing (from the PRISMA 2020 statement) item. We follow the order of the PRISMA 2020 statement (ie, we present the items numerically from 1 to 27). However, similarly to the PRISMA 2020 statement, authors do not need to follow the same order in their reports. Rather, what is important is that the different items and elements are addressed somewhere within the report.

Then, and for each of the PRISMA-LSR checklist items and elements, we provide an 'explanation' section where we provide the rationale for including them, and we cite literature to justify their inclusion. Furthermore, for each item and element, we provide examples of complete reporting. We edited some examples by removing citations or by spelling out abbreviations. The examples were identified by the authors of the PRISMA-LSR extension.

We also include a section about the LSR status with examples of good reporting practice.

During the development of this extension, the expert panel identified issues that they considered relevant to the reporting of LSRs but which we judged as more related to the publishing of LSRs. That judgment considered whether the issue is the responsibility of the authors (reporting-related) or that of the editors or publishers (publishing-related). We list those publishing issues at the end of the document.

Journals and publishers might impose limits (eg, on wording and on the number of tables and figures allowed in the LSR report). In such instances, if the relevant information for some items or elements already appears in a publicly accessible document (ie, LSR protocol, previous LSR

version), referring to that document may suffice. Alternatively, we recommend reporting on that information in supplementary files, which ideally should be publicly accessible.

2. Explanation and examples for the PRISMA-LSR checklist

Title

PRISMA 2020 item 1: Identify the report as a systematic review.

- **PRISMA-LSR element: Identify the report as "living" in the title.**

Explanation
Including the term 'living' in the title allows readers to directly recognize that the systematic review uses the living mode. It also allows appropriate indexing in databases. We have chosen the term 'living' as opposed to 'up to date', or 'continuously updated', given that the term is widely used. Note that while PRISMA 2020 item 1 included an additional (ie, non-essential) element to report whether "the review is a continually updated ('living') systematic review," in PRISMA-LSR, this is an essential element.
When the authors stop maintaining a systematic review in the living mode, they should cease including the term living in reports of subsequent versions. Previous publications would still have the label of 'living' given they were conducted in the living mode when published.

Examples
1. "Interleukin-6 blocking agents for treating COVID-19: a living systematic review"[9]
2. "Drug treatments for covid-19: living systematic review and network meta-analysis"[10]

- **PRISMA-LSR element: Provide the version number.**

Explanation
Since for each update of the LSR a new version is created, providing the version number in the title will help readers easily identify what version of the LSR they are accessing. The first version of an LSR has been also referred to as the 'base' version. Using the term 'version' allows the use of only one term, which is simpler than using two terms ('base' and 'update'). The reporting of an LSR protocol should follow the PRISMA for Protocols (PRISMA-P) 2015 statement (currently being updated[11]), as well as address specific PRISMA-LSR items. These are PRISMA-LSR items 1 (ie, title item), 3-15, and L1 (ie, all introduction and methods items except item L2, which addresses changes to the methods). We do not address including the date of publication of the version in the title as this is a publishing aspect and should be obvious from the publication itself.

Examples
1. "Zika virus infection as a cause of congenital brain abnormalities and Guillain-Barré syndrome: A living systematic review [version 1; peer review: 2 approved]"[12]
2. "SARS-CoV-2 transmission in schools: An updated living systematic review (version 2; November 2020)"[13]

Abstract

PRISMA 2020 item 2: See the PRISMA 2020 for Abstracts checklist.

- **PRISMA-LSR element: Indicate whether the LSR is being retired from the living mode after the publication of the current version, if applicable.**

Explanation
When applicable, including plans to retire the LSR will inform the reader that another version of the LSR is not to be expected. See glossary of the PRISMA-LSR document for a definition of the term “retirement from the living mode”. Refer to the elements about retirement below for more details about retiring LSRs (third PRISMA-LSR element of item L1 and third PRISMA-LSR element of item 23d).

> *Example*
> “This fifth quarterly update including randomized controlled trials published through 19 October 2021 is the final update for this living review according to the preplanned protocol.”[14]

Introduction

Rationale

PRISMA 2020 item 3: Describe the rationale for the review in the context of existing knowledge

- **PRISMA-LSR element: Justify the use of the living mode.**

Explanation
Since not all topics would qualify for the living mode, readers need to understand why the living mode is needed to address a certain topic. They also need to understand why authors continue to use the living mode in subsequent versions of the review. Three criteria have been proposed to guide the decision to initiate an LSR and can be used by LSR authors to justify the adoption or continuation of the use of the living mode. Specifically, authors could indicate (1) how a systematic review is a priority for decision-making, (2) that the certainty in the existing evidence is low or very low, and (3) that there is likely to be new research evidence.[15] This justification would also capture any request from decision makers to make the review an LSR.

> *Examples*
> 1. “We performed this review as a living systematic review because research into many aspects of Zika virus is a new and fast-moving field. Several studies are ongoing and have published interim results, and updated results could affect public health decisions.”[16]
> 2. “The research questions in this systematic review are in line with proposed criteria for continuing a living systematic review, namely: (1) the systematic review is a priority for decision making; (2) new information will change decision making; and (3) there is

likely to be, on an ongoing basis, new research relevant to decision making. As such, if these criteria are still met at the conclusion of our baseline review and analysis, we will convert the review to a living systematic review with network meta-analysis. Given the number of new systemic medications in development for atopic dermatitis, this is likely to be the case."[17]

3. "We plan to maintain this review as a living systematic review. […] This approach is appropriate for this review since it addresses an important clinical topic and there is currently significant uncertainty as to the most effective intervention. It is therefore important that consumers and healthcare providers have access to the most up-to-date evidence to make informed decisions. The review authors are aware of several relevant ongoing trials that will be important to incorporate in a timely manner."[18]

- **PRISMA-LSR element: Cite the preceding version of the LSR, if applicable.**

Explanation

Citing the preceding version of the LSR (if one exists) can help readers easily determine where to locate that version if they are keen to examine it. A methodological survey of 76 LSRs examined access to the preceding LSR version from an LSR update version. Access for partial reports was as follows: accessible within the document (66.4%), link to supplementary file in the update version (11.8%), citation (0%), and not accessible (21.8%).[19] Access for full reports was as follows: accessible within the document (22.9%), link to supplementary file in the update version (51.4%), citation (0%), not accessible (20%), and other (5.7%).[19] Authors could provide a reference, the doi, or link to the preceding version.

In addition to linking to the preceding version, linkages could be provided to all previous versions of the LSR.

Examples

1. "This is the fifth version of the original article published on 30 July 2020 (reference), and previous versions can be found as data supplements."[10]
2. "This is the fifth update of our living, rapid review on remdesivir for adults with COVID-19 (reference). [...] Our first update, which included randomized controlled trials published through 7 December 2020 (references), led to a major update (reference). Our second update, including randomized controlled trials published through 8 February 2021, found no new evidence (reference). Our third update (reference) derived from randomized controlled trials published through 10 May 2021 included 1 new randomized controlled trial (reference), and our fourth update of randomized controlled trials published through 9 August 2021 (reference) included 1 new add-on subtrial of the World Health Organization Solidarity trial—the Norwegian Solidarity trial (reference)."[14]

Objectives

PRISMA 2020 item 4: Provide an explicit statement of the objective(s) or question(s) the review addresses.

- **PRISMA-LSR element: Describe and justify any changes since the preceding version to the review's objective(s) or question(s).**

Explanation
During the course of an LSR, the elements of the review question (eg, population, interventions, and outcomes) might change; either existing ones become irrelevant or new ones emerge. For example, a medication may no longer be licensed for use, or the understanding of the condition being studied may evolve. Indeed, one study found that the interventions and comparators assessed by LSRs in the COVID-19 pandemic evolved over time and needed adaptations. Also, outcome measures were likely to change as more evidence became available, and as standard reporting measures changed over time [20]. Hence, it would be important not only to describe but also to justify changes to the review's objective(s) or question(s). Changes to the review's objective(s) or question(s) include any change in at least one of the PICO elements. The extension requires the reporting of only changes since the preceding version (or the latest mention of the items or element in question if not included in the preceding version, eg, when that version is a partial report). LSR authors could, in addition, report on changes since the protocol.

> *Example*
> “We added the outcome 'quality of life' after discussion with a patient representative.”[21]

Methods

Living mode parameters

PRISMA-LSR item L1: Specify the living mode parameters.
Living mode parameters can be placed in a box to make them more prominent. Box 2 of the PRISMA-LSR publication gives a definition of the term “living mode parameters.”

- **PRISMA-LSR element: Specify the planned schedule of the search for each source (eg, at a prespecified interval, following predefined triggers).**

Explanation
LSRs are characterized by frequent updates to the literature search, which can be done following different schedules[22] (eg, fixed-interval schedule, following pre-defined triggers). Pre-defined triggers could include LSR authors becoming aware of the publication of an eligible study (eg, through communication with colleagues, through a press release). Examples of schedules also include automatic alerts and monitoring study registers. A methodological survey of 76 LSRs found that 33 LSRs (43%) reported a fixed-interval schedule for updating; the median and IQR of the planned period of update being 3 months and 1-4.5 months.[23] Most COVID-19 related LSRs considered by a concept paper addressing methodological challenges for LSRs ran their search weekly or monthly.[20] The planned schedule of search is important to report so that users can assess the adequacy of the search update approach and understand how frequently the review will be updated. The planned schedule could be based on a specific rationale or simply on convenience.

Example<br>"An automated search is run every day, with results deduplicated and imported into Research Electronic Data Capture."[16]

- **PRISMA-LSR element: Specify the planned schedules for the remaining steps of the systematic review (eg, at a prespecified interval, following predefined triggers), if applicable. The remaining steps include screening, data collection, risk of bias assessment, analysis, certainty of evidence assessment, and publication.**

Explanation

The planned schedules for the remaining steps of the systematic review could be the same as those of the search, or different. For example, a scoping review of the methodological literature and guidance on how to conduct, report, publish and appraise the quality of LSRs found that the frequency for data abstraction could be determined (1) by the continuous search (trigger dependent), (2) immediately after study identification, or (3) once new evidence has been identified for inclusion. Similarly, for quality and risk of bias assessment, updating could (1) be regular, at a defined time interval, or (2) occur once new evidence has been identified for inclusion. For data synthesis, updating could occur (1) immediately after new study inclusion, (2) on a continuous basis, (3) once new evidence has been identified for inclusion. Only one paper identified by that scoping review reported on the frequency of certainty of evidence assessment, which was following regular updating.[24] Details about the planned schedules for the different steps of the systematic review are important report so that users can assess the adequacy of methods and understand how frequently the review will be updated. The planned schedule could be based on a specific rationale or simply on convenience. Authors might decide to prioritize certain outcomes or comparisons to be updated more frequently than others. In that case, they could report on the different updating frequencies.

Example<br>"Our aim is to update the synthesis at least once every week. For this purpose, we will search for, screen and extract data every day. The updated synthesis will be reported online at least once every week. In addition, we will update this Cochrane Review at least once every six months, or as soon as the certainty of evidence (assessed with the GRADE methodology) changes. We will wait until the accumulating evidence changes one or more of the following aspects of the review, before incorporating it and re-publishing the Cochrane Review:<br>a. the findings of one or more critical outcomes;<br>b. the credibility (eg, GRADE rating) of one or more critical outcomes;<br>c. new settings, population, interventions, comparisons or outcomes studied; or new serious adverse events."[25]

- **PRISMA-LSR element: Specify the plan for retirement from the living mode (eg, based on a prespecified timeline, following predefined triggers), if there is one. If there is no such plan, indicate so.**

Explanation

When applicable, specifying plans for retiring the LSR from the living mode is important so that users can assess the adequacy of the plan and understand when updates are not to be expected anymore.
This element recommends reporting the general plan for retirement from the living mode (see glossary), while the third PRISMA-LSR element of item 23d recommends reporting the reason for retiring the LSR in question. Murad et al. proposed a number of triggers that may lead to an LSR being retired. These include: (1) when the evidence becomes conclusive, which can be determined based on certainty of the evidence or statistical methods, (2) when the topic becomes less relevant to stakeholders, (3) when new studies are not expected to be published, (4) when required resources become unavailable;[26] however, other triggers may also be possible.

Examples

1. "We plan to update our literature search […] every 2 months through December 2021[…]."[27]
2. "Each year, we will consider the necessity for the review to be a living systematic review by assessing ongoing relevance of the question to decision-makers and by determining whether uncertainty is ongoing in the evidence and whether further relevant research is likely."[18]

Data collection process

PRISMA 2020 item 9: Specify the methods used to collect data from reports, including how many reviewers collected data from each report, whether they worked independently, any processes for obtaining or confirming data from study investigators, and if applicable, details of automation tools used in the process.

- **PRISMA-LSR element: Describe whether the review team updated or planned to update collected data for a previously included study when relevant.**

Explanation
Information from a previously included study might be subject to change based on newly available reports, or based on personal communication between LSR authors and primary study authors. Hence, it would be important for LSR authors to report on the plans to update data for a previously included study when these data change, or on actual updates of the data if new information for a previously included study is obtained for the current version of the LSR. Newly available reports do not only refer to peer reviewed publications, but also to preprints, grey literature (eg, regulatory report submitted to governmental agency), a web page, etc. Examples of changes include data from a preprint article changing following peer review, and data about a certain outcome changing in a new report of a study with a longer follow up time or with a larger sample size. Indeed, a cross-sectional study of preprints and final journal publications found that 22% of studies had at least one outcome that was included in the journal publication, but not the preprint, while 12% of studies had at least one outcome that was reported in the preprint only. The study also found that common discrepancies between preprints and final journal publications were differences in numerical values and statistical significance, additional statistical tests and subgroup analyses, and longer follow-up times for outcome assessment in journal publications.[28]

Examples

1. "Three out of 98 included studies (3%) were only available as preprints at the time of the search. We will update data extracted from these studies in future versions of our review as these studies become published in peer-reviewed journals."[29]
2. "We track preprints of randomized controlled trials until publication and update data to match that in the peer reviewed publication when discrepant, and reconcile corrections and retractions if they exist."[30]

Study risk of bias assessment

PRISMA 2020 item 11: Specify the methods used to assess risk of bias in the included studies, including details of the tool(s) used, how many reviewers assessed each study and whether they worked independently, and if applicable, details of automation tools used in the process

- **PRISMA-LSR element: Describe whether the review team updated or planned to update risk of bias information for a previously included study when relevant.**

Explanation

Information from a previously included study might be subject to change based on newly available reports, or based on personal communication between LSR authors and primary study authors. Hence, it would be important for LSR authors to report on the plans to update risk of bias information for a previously included study when this information changes, or on actual updates of the risk of bias information if new information for a previously included study is obtained for the current version of the LSR. Newly available reports do not only refer to peer reviewed publications, but also to preprints, grey literature (eg, regulatory report submitted to governmental agency), a web page, etc.

Example

"Some data are included from preprints, and these might be subject to change following peer review. Therefore, some results, bias risk assessments, and GRADE summaries might change in later editions of this living systematic review following inclusion of the published peer-reviewed manuscripts."[31]

Synthesis methods

PRISMA 2020 item 13d: Describe any methods used to synthesize results and provide a rationale for the choice(s). If meta-analysis was performed, describe the model(s), method(s) to identify the presence and extent of statistical heterogeneity, and software package(s) used.

- **PRISMA-LSR element: Report any analytical methods applied specifically because of the living mode.**

Explanation

Analytical methods applied specifically because of the living mode have been proposed (eg, for correcting type 1 error)[32] and LSR authors might decide to use them. Since there is no consensus

on when these analytical methods should be used, it would be important for LSR authors to report on any plans to use them. This extension does not endorse any specific analytical method.

Examples
1. "If required, meta-analyses will be updated by use of trial sequential analyses (type I error, 0.05; power, 80%; assumed effect size, moderate Cohen's effect size [0.5 standard deviations]) for living systematic reviews."[33]<br>2. "We will not use formal sequential meta-analysis approaches for updated meta-analyses."[18]

Changes to the methods

PRISMA-LSR item L2: Describe changes to the methods.

This item replaces the third and fourth elements of item 10a, and item 24c of the PRISMA 2020 checklist.

- **PRISMA-LSR element: Describe and justify any changes since the preceding version to the methods (items L1, 5-15).**

Explanation

The methods of an LSR may change across versions. Reasons for change could relate to the LSR question (eg, evolving understanding of the condition being studied) or to the LSR processes (eg, emergence of new tools). Such changes are important to report and justify. It might be too cumbersome for readers, and burdensome for authors, to report both changes since the preceding version and (cumulative) changes since the publication of the protocol. Therefore, the extension recommends the former. LSR authors could, in addition, report on changes since the protocol. If authors decide to consolidate the changes to the methods in a section of the LSR, they would indicate in the PRISMA-LSR checklist the page(s) where the consolidated section is included. If authors decide to report changes within the relevant section of the LSR report, they would indicate in the PRISMA-LSR checklist the page(s) where the relevant methods sub-sections are included.

Examples of changes to the methods that could arise (identified by the expert panel) include:

- Relating to PRISMA 2020 item 5: Authors should always rely on the best available evidence, which will likely evolve and change rapidly over time.[20] Hence, eligibility criteria (eg, in terms of study design, types of publication) might change over the course of the LSR. For example, for some COVID-19 related LSR, authors initially included non-randomized studies, but later on excluded those and included clinical trials.[20]
- Relating to PRISMA 2020 item 6: Databases may be discontinued or no longer be accessible, and new information sources could be added. Indeed, a challenge reported for reviews conducted during the COVID-19 pandemic was the dynamic nature of electronic databases.[20] For example, the Centers for Disease Control and Prevention COVID-19 Research Articles Downloadable Database, an early and comprehensive source of pre-print articles, was discontinued in mid-2020, but was later completely covered by the World Health Organization COVID-19 Global literature on coronavirus disease database.[20] Refer to PRISMA-S for reporting on the search process.[34]

- Relating to PRISMA 2020 item 7: As the understanding of the condition being studied and the evidence evolves, relevant terms, keywords or database filters may change. According to the 2019 Cochrane guidance for LSRs, the search strategies need to be updated.[22] We have kept it flexible for the authors to choose where to report the changes to the search strategies (eg, in the main text, in the appendix). The PRISMA 2020 checklist requires the reporting of the search strategy, i.e., the latest search strategy (possibly modified from the preceding version) will be reported. The PRISMA-LSR extension requires the reporting of any modifications and their justifications. Previous versions of the search will be included in previous versions of the LSR and the readers can refer to these. The second PRISMA-LSR element of item 3 would facilitate the access of readers to the earlier version of the search strategy. For the search strategy, consider reporting only on "important" changes. Examples include use of a new search filter and removing a search block to increase sensitivity. Refer to PRISMA-S for reporting on the search strategies.[34]
- Relating to PRISMA 2020 item 8: To enhance efficiency during the course of the LSR, authors might apply changes to their screening process. Changes to this item include changes to any aspect of the screening process such as the application of machine learning/automation tool or the use of crowdsourcing.
- Relating to PRISMA 2020 item 9: To enhance efficiency during the course of the LSR, authors might apply changes to their data abstraction process. Changes to this item include changes to any aspect of the data abstraction process such as the application of machine learning/automation tool or the use of crowdsourcing.
- Relating to PRISMA 2020 item 10: New outcomes may be added in subsequent versions of an LSR with emerging information about new outcomes (eg, COVID vaccines and vaccine-induced immune thrombotic thrombocytopenia). Changes in outcomes include the measurement of an outcome at a new follow-up time point, and modification of a core outcome set. Authors may cease to update the analyses for a specific outcome in the living mode.
- Relating to PRISMA 2020 item 11: As eligibility by study design might change during the course of the LSR, authors should report on changes in the risk of bias assessment tool(s) used. To enhance efficiency during the course of the LSR, authors might apply changes to their risk of bias assessment process. Changes to the latter include changes in the application of machine learning/automation tool or the use of crowdsourcing.
- Relating to PRISMA 2020 item 12: Effect measures may be added or changed (eg, risk ratio to hazard ratio), for example, when new studies become available. Changes to this item also include a change in thresholds used to interpret the size of effect.
- Relating to PRISMA 2020 item 13: As the evidence evolves, authors might decide to plan for new analyses.
- Relating to PRISMA 2020 item 15: As the evidence evolves, authors might decide to change the list of comparisons and outcomes subject to certainty assessment.

Example relating to PRISMA-LSR item L1
"Since August 2021, we have run the searches monthly instead of weekly."[21]

Example relating to PRISMA 2020 item 5

"We amended eligibility criteria after the third version of the review (reference) in 2 ways. First, we excluded studies that only reported the proportion of presymptomatic SARS-CoV-2 because the settings and methods of these studies were very different and their results were too heterogeneous to summarise (reference) […]"[35]

Example relating to PRISMA 2020 item 6
"We stopped searching the Chinese databases on 20 February 2021 because they had not provided studies that meaningfully altered the evidence for any intervention."[10]

Example relating to PRISMA 2020 item 7
"At the beginning of 2021, new MeSH or EMTREE terms were inserted in Medline and Embase, so the whole search strategies were revised and new search terms like IGY-110 or GIGA-2050 or GC5131 or 5131A or INOSARS were added."[21]

Example relating to PRISMA 2020 item 8
"Due to the increased volume of published and preprint articles, we used artificial intelligence text analysis from 25 May 2020 and onwards to conduct an initial classification of documents, based on their title and abstract information, for relevant and irrelevant documents."[29]

Example relating to PRISMA 2020 item 9
"We had planned to extract data using a standardised data extraction form developed in Covidence. However, we could not adapt the standardised form to our needs. Therefore we generated a customised data extraction form in Microsoft Excel (Microsoft Corporation 2018)."[21]

Example relating to PRISMA 2020 item 10
"We renamed the outcome 'time to discharge from hospital' to 'Duration of hospitalisation, or time to discharge from hospital' to clarify that we are interested in both, continuous and time-to-event data."[21]

Example relating to PRISMA 2020 item 11
"After the third version of the review (reference), we developed a new tool to assess the risk of bias because the study designs of included studies have changed."[35]

Example relating to PRISMA 2020 item 13
"We had added subgroup analyses for the following characteristics in this update of the review.

- Duration since symptom onset
- Level of antibody titre in donors
- Level of antibody titre in recipients at baseline
- SARS-CoV-2 variants

Considering the currently available evidence, we decided to add these subgroups, because their role in the effectiveness of convalescent plasma is currently being discussed and needs to be further investigated."[21]

Example relating to PRISMA 2020 item 14

"In case of missing data, we conducted an available-case analysis" (under differences between fourth and current published review version).[21]

Example relating to PRISMA 2020 item 15
"At protocol stage, we had planned to assess the certainty in the evidence for our primary outcomes (all-cause mortality at hospital discharge and time to death) only. However, as none of the included studies reported any deaths during their study periods, we decided to assess the certainty in the evidence also for prioritised secondary outcomes (clinical improvement, grades 3 and 4 adverse events, and serious adverse events) to increase the informative value on effectiveness and safety of convalescent plasma therapy."[21]

- **PRISMA-LSR element: If there are no changes to the methods, indicate so.**

Explanation
Providing a general statement about the absence of any changes to the methods, or reporting that particular methods did not change indicates to returning readers that they may not need to re-read the particular methods section.

Example
"Tools to assess risk of bias and estimate certainty of evidence (COE) were unchanged."[14]

- **PRISMA-LSR element: Indicate whether the changes to the methods were applied to previously included studies.**

Explanation
It is possible for changes to the methods of an LSR to be consequential on previously included studies. Hence, authors should indicate whether the changes to the methods were applied to previously included studies (eg, repeating risk of bias assessment for a previously included study based on the use of a new risk of bias assessment tool). When changes were not applied to previously included studies, this should be reported.

Examples
No examples found.

Results

Study selection

PRISMA 2020 item 16a: Describe the results of the search and selection process, from the number of records identified in the search to the number of studies included in the review, ideally using a flow diagram.

- **PRISMA-LSR element: Describe what triggered the current version, if applicable.**

Explanation

Since the schedule for updating an LSR could be according to a pre-specified interval, or following pre-defined triggers, when the latter case applies, authors should indicate the actual trigger for the current LSR version. Triggers include becoming aware of a newly published eligible study, access to previously unavailable data, and a request by the commissioner of the review to update its findings.

Example

Reis S. et al. maintain an excel spreadsheet that is publicly accessible on the Open Science Framework (OSF) platform and that details the living evidence surveillance process. In this spreadsheet, authors highlight the study triggering the republication of the review (see last column in figure 1 below).[1-3]

Date of last search	# records received		# records deduplicated	# records screened		Categorisation of reports included at fulltext screening								Trigger to publish update
	Total records from databas e search	Total records database + other sources	Total after deduplication in EndNote	title/ abstract	fulltext	# included studies	# reports of included studies	# ongoing studies	# reports of ongoing studies	# studies awaiting classifica tion	# reports of studies awaitin g	# reports excluded	# additional manual deduplica tion	
11/04/2022	81	81	60	60	13	1	4	2	6	0	0	3	0	yes (EPIC-HR
11/05/2022	161	161	74	74	7	0	0	4	4	0	0	3	0	no
10/06/2022	187	187	21	21	2	0	0	2	2	0	0	0	0	no
11/07/2022	217	217	22	22	1	0	0	0	0	0	0	1	0	no
11/08/2022	259	259	27	27	6	0	0	0	0	0	0	6	0	no
12/09/2022	288	288	35	35	7	0	0	0	0	0	0	6	1	no
13/10/2022	335	335	32	32	12	0	0	3	3	0	0	9	0	no
11/11/2022	383	383	34	34	5	0	0	1	2	0	0	3	0	no
15/12/2022	429	429	41	41	6	0	0	2	2	1	1	3	0	no
18/01/2023	491	491	41	41	8	0	1	1	3	0	0	2	2	no
16/03/2023	617	618	113	113	12	1	4	-3	-5	0	1	12	0	yes (Liu-2023)
15/05/2023	739	739	93	93	17	0	0	1	-2	2	6	13	1	no
21/07/2023	834	834	108	108	7	0	0	5	5	0	0	2	0	no
15/09/2023	940	940	102	102	6	0	0	-1	-1	0	0	6	0	no
15/11/2023														
Total	940	940	803	803	109	2	9	17	19	3	8	69	4	

Figure 1. Snapshot of excel spreadsheet detailing the living evidence surveillance process for the review by Reis et al. (some columns have been deleted for clarity of presentation) [1-3].

- **PRISMA-LSR element: Ideally, use a flow diagram to illustrate the results of the search and selection processes in the different versions of the review using one of the LSR tailored flow diagrams.**

Explanation

Returning readers of the LSR might be most interested in knowing the results of the search and selection processes since the preceding version. New readers of the LSR might be most interested in knowing these results since the start of the review. That is why the PRISMA-LSR extension proposes four approaches to presenting the flow diagram (see below). A methodological survey of 76 LSRs found that 96% of base versions and 93% of full updates used a flow diagram to report on the search results, whereas only one partial update presented a flow diagram. Among LSR update versions., 21% reported the search results for the base version and for each update version separately, 11% reported on the search results for the base version separately and for all update versions combined, 13% reported the search results for the latest update version separately and for all previous versions combined, 6% reported the search results for all the different versions combined, 26% reported the search results for the latest update version only, and 23% did not report the search results at all.[19] This element replaces the second

element of item 16a of the PRISMA 2020 checklist. The approaches proposed in the PRISMA-LSR extension based on the study by Kahale et al. are as follows:[19]

- Approach 1: presenting the search results of the different versions separately (ie, first (base) version and each update separately);
- Approach 2: presenting the search results for the different versions combined (ie, including first version and all update versions);
- Approach 3: presenting the search results for the first version separately, and the results of all update version combined;
- Approach 4: presenting the results of the latest update version separately, and the results of all previous versions (including the first version) combined.

For documenting the LSR study flow, Kahale et al. propose using a spreadsheet for one LSR at a time, consisting of tabs for each of the respective search sources and a final 'cumulative' tab that keeps track of all records [19]. Refer to PRISMA-S for reporting on the search results.[34] Refer to the R package (https://github.com/nealhaddaway/livingPRISMAflow) and web-based ShinyApp (https://estech.shinyapps.io/livingprismaflow/) to create a PRISMA 2020 flow diagram for LSRs.[36] Authors could point to studies expected to be published by the planned next version and consequently included.

> _Examples_
> 1. "Supplement Figure 1 displays the PRISMA (Preferred Reporting Items for Systematic reviews and Meta-Analyses) flow diagram for living systematic reviews."[37]
> 2. "We reported the process in a flow diagram, adapted for living systematic reviews (reference to figure)."[35]

Results of syntheses

PRISMA 2020 item 20b: Present results of all statistical syntheses conducted. If meta-analysis was done, present for each the summary estimate and its precision (eg, confidence/credible interval) and measures of statistical heterogeneity. If comparing groups, describe the direction of the effect.

- **PRISMA-LSR element: Report results of any analytical methods applied specifically because of the living mode.**

Explanation
This item relates to the PRISMA-LSR element of item 13d (methods section). As previously indicated, this extension does not endorse any specific analytical method, and recognizes that there is no consensus about when these analytical methods should be used.

> _Example_
> "The trial sequential analysis showed that we had enough information to reject that lopinavir-ritonavir versus control reduces the risk of all-cause mortality with a relative risk reduction of 20%."[38]

Changes to the results

PRISMA-LSR item L3: Describe changes to the results.

In general, highlighting changes to the results is particularly helpful for returning readers. Examples of what could cause changes to the results include the inclusion of new studies, changes related to the already included studies (eg, retraction of a study, obtaining new information from study authors), or changes in the LSR methods (eg, use of a new risk of bias tool). When the reason for changes to the results is not obvious, it is important to describe the reason for the changes. It might be too cumbersome for readers, and burdensome for authors, to report both changes since the preceding version and (cumulative) changes since the publication of the protocol. Therefore, the extension recommends the former. LSR authors could, in addition, report on changes since the protocol. If authors decide to consolidate the changes to the results in a section of the LSR, they would indicate in the PRISMA-LSR checklist the page(s) where the consolidated section is included. If authors decide to report changes within the relevant section of the LSR report, they would indicate in the PRISMA-LSR checklist the page(s) where the relevant results sub-sections are included.

- **PRISMA-LSR element: Indicate the studies that were included since the preceding version (related to PRISMA 2020 item 17).**

<u>*Explanation*</u>

Indicating the studies that were included since the preceding version should be reported. Highlighting these studies may decrease the burden for returning readers. This element applies to both studies and reports of studies.

<u>*Example*</u><br>“We included one study for outpatients and one study for inpatients in our qualitative synthesis. One study was in the original review (reference), and one study from this update (reference).”[39]

- **PRISMA-LSR element: Describe and justify the changes since the preceding version in the eligibility status of any study (ie, excluding a previously included study, including a previously excluded study; related to PRISMA 2020 item 16).**

<u>*Explanation*</u>

It is important for readers to be aware of any changes to the eligibility status of studies and of the reasons for the changes. The justification could be related to a change in LSR methods (eg, eligibility), or to obtaining new information from newly available reports (including withdrawal or retraction of a study, expression of concerns) or from personal contact with authors. Newly available reports refer to peer reviewed publications, preprints, grey literature, a web page, etc. The status of studies labeled as “ongoing” and “awaiting classification” in the preceding LSR version should be updated, if applicable.

<u>*Example*</u><br>“Since the first iteration, one trial addressing ivermectin and showing large positive effects was retracted. The living nature of our systematic review and network meta-analysis enables the exclusion of retracted data from this second iteration and between subsequent iterations if needed.”[40]

- **PRISMA-LSR element: Describe any other consequential changes since the preceding version to the results.**

Explanation

Reporting consequential changes to the results is recommended. These include changes that are likely to impact the interpretation of the effect size (eg, small to moderate effect size), and the certainty assessment. LSR authors could, in addition, report on any changes to the results.

Examples of changes to the results that could arise (identified by the expert panel) include:

- Relating to PRISMA 2020 item 17: Characteristics of a previously included study might be subject to change based on newly available reports, including errata. Newly available reports refer to peer reviewed publications, preprints, grey literature, a web page, etc. A specific example is platform trials (ie, “trials that study multiple targeted therapies in the context of a single disease in a perpetual manner, with therapies allowed to enter or leave the platform on the basis of a decision algorithm”[41]), which might include new interventions between review updates, or might conduct a nested study within a different original study design. In these cases, authors would report on a change in study design or study intervention. Updating the primary references of already included studies is another example of a change worth documenting.
- Relating to PRISMA 2020 items 18 and 21: Consequential changes could be related to a change in the risk of bias tool used by the authors, to obtaining new information from newly available reports (eg, checking whether data previously abstracted from a preprint have changed in the peer reviewed paper, abstracting new data) or from personal contact with authors. Newly available reports refer to peer reviewed publications, preprints, grey literature, a web page, etc.
- Relating to PRISMA 2020 item 20b: Indicate which outcomes have results of statistical syntheses available for the first time. This includes a measurement of an outcome at a new follow-up time point. Indicate syntheses that have been removed since the preceding version.
- Relating to PRISMA 2020 item 20c: Indicate outcomes for which investigations of possible causes of heterogeneity have results available for the first time. Indicate investigations of possible causes of heterogeneity that have been removed since the preceding version.
- Relating to PRISMA 2020 item 20d: Indicate outcomes for which results of sensitivity analyses are available for the first time. Indicate sensitivity analyses that have been removed since the preceding version.
- Relating to PRISMA 2020 item 22: Change in certainty of evidence should be reported when applicable for each outcome. Refer to detailed GRADE guidance for explanatory footnotes to support changes in the GRADE certainty in the evidence judgments.[42]

Example relating to PRISMA 2020 item 17

“Twelve preprints were subsequently published after peer review. The supplementary data present the differences between study preprint and peer-reviewed publications.”[30]

Example relating to PRISMA 2020 item 18

"Published reports of 3 studies previously available as preprints became available (references), enabling more thorough assessment for risk of bias. The risk of bias is now determined to be serious for Yu and colleagues' study, remains high for Tang and colleagues' study, and changed from moderate to serious for Mahévas and colleagues' study."[43]

Example relating to PRISMA 2020 item 20b
"Figure 16 displays the pooled sensitivity and specificity estimates with 95% confidence intervals from all four versions of this review (ie, Salameh 2020a published in September 2020, Islam 2020 published in November 2020, Islam 2021 published in March 2021, and this current version). The sensitivity estimates of chest CT appear to be similar across McInnes 2020, Islam 2020, Islam 2021 and this current version, while the specificity estimates of chest CT appear to increase from Salameh 2020a to Islam 2021, and then remain similar between version 3 and the current version."[29]

Example relating to PRISMA 2020 item 20c
"The correction of analysis 17.2 changed the conclusion for the subgroup analysis age of participants, as the test for subgroup differences was not significant anymore."[21]

Example relating to PRISMA 2020 item 22
"The newly included randomized controlled trials strengthen previous findings on the benefit of remdesivir on the proportion of patients receiving ventilation or extracorporeal membrane oxygenation at follow-up but decreases the strength of previous findings on the reduction of serious adverse events with remdesivir."[14]

- **PRISMA-LSR element: If there are no changes to the results, indicate so.**

Explanation
Providing a general statement about the absence of any changes to the results, or reporting that particular results did not change indicates to returning readers that they may not need to re-read the particular results section.

Example
"Overall, the addition of the new studies and the retraction of 1 prior study does not change the findings or certainty of evidence ratings we reported in the original review."[44]

Discussion

PRISMA 2020 item 23a: Provide a general interpretation of the results in the context of other evidence.

No new elements were identified.

PRISMA 2020 item 23b: Discuss any limitations of the evidence included in the review.

No new elements were identified.

PRISMA 2020 item 23c: Discuss any limitations of the review processes used.

- **PRISMA-LSR element: Discuss any limitations related to the living mode.**

<u>*Explanation*</u>
While the main advantage of the living mode is to produce up-to-date evidence, it has its own limitations. Potential limitations include time lag bias, inability to meet update schedule, inability to include eligible data because of pressure to meet update schedule, and increased risk of false positive findings related to frequent updates of the meta-analyses. Discussing these limitations is important for readers to assess the trustworthiness of the review findings.

> <u>*Example*</u>
> "The living nature of our systematic review and network meta-analysis could amplify publication bias, because studies with promising results are more likely to be published and are published sooner than studies with negative results. Given the failure of hydroxychloroquine trials to show benefit, this is not a concern for hydroxychloroquine."[40]

PRISMA 2020 item 23d: Discuss implications of the results for practice, policy, and future research.

- **PRISMA-LSR element: Describe any changes since the preceding version to the implications of the results for practice, policy, and future research.**

<u>*Explanation*</u>
While changes to the findings of an LSR could be minor, some changes could be consequential with implications on practice, policy, and future research, and therefore important to highlight. The extension requires the reporting of only changes since the preceding version (or the latest mention of the items or element in question if not included in the preceding version, eg, when that version is a partial report). LSR authors could, in addition, report on changes since the protocol.

> <u>*Examples*</u>
> 1. "It is becoming increasingly unlikely that in-hospital use of hydroxychloroquine will yield beneficial effects."[45]
> 2. "The substantive new evidence changes the nature and strength of our earlier conclusions."[46]
> 3. "New information allowed us to assess the effect of remdesivir on mortality in clinically meaningful subgroups."[46]

- **PRISMA-LSR element: Describe and justify any planned changes to review methods in upcoming review versions.**

<u>*Explanation*</u>
One advantage of use of the living mode is the ability to tailor the methods of the LSR according to the emergence of new methods, or to changes in the context (eg, new population that needs to

be addressed, emergence of new tools for the conduct of LSRs, a decrease in the resources of the LSR authors). Describing these changes is important so that readers can assess the adequacy of the new review methods and understand what to expect.

Example
"Our future updates will also focus on studies investigating suicide deaths, suicide attempts and self-harm. We will no longer include studies: with suicidal thoughts and "suicide risk" as outcomes; modelling studies (since these have been superseded by studies based on suicide deaths) and those based on social media posts (because of the lack of evidence for diagnoses and self-selecting biases in respect of who contributes to these)."[47]

- **PRISMA-LSR element: Indicate and justify whether the LSR is being retired from the living mode following the publication of the current version, if applicable.**

Explanation
When applicable, including plans to retire the LSR will inform the reader that another version of the LSR is not to be expected. The justification (eg, reaching high certainty evidence) would also be informative for the readers. See glossary of the PRISMA-LSR document for a definition of the term "retirement from the living mode". A methodological survey of 76 LSRs found that none of the included LSRs that had a period since the last published version greater than 3 times the planned period of update included a "retirement notice" in their latest version.[19] The third element of item L1 reflects general plans for retirement from the living mode (eg, following predefined triggers), while this element reflects an actual decision to retire the LSR in question.

Examples
1. "Considering that current research is exploring ICI in association with chemotherapy or other immunotherapeutics drugs versus ICI as single agent rather than platinum based chemotherapy, we believe that platinum based chemotherapy alone will be no longer the proper standard arm of future randomised clinical trials in this setting. For this reason, this review has been transitioned out of living mode and will, from now on, be updated on a 2-years basis like any other Cochrane systematic review."[48]
2. "The review authors have decided to cease maintaining this review in living systematic mode as a reasonable level of certainty has been reached in the existing evidence."[49]

Other information

Registration and protocol

PRISMA 2020 item 24a: Provide registration information for the review, including register name and registration number, or state that the review was not registered.

No new elements were identified.

PRISMA 2020 item 24b: Indicate where the review protocol can be accessed, or state that a protocol was not prepared.

No new elements were identified.

Support

PRISMA 2020 item 25: Describe sources of financial or non-financial support for the review, and the role of the funders or sponsors in the review.

- **PRISMA-LSR element: Describe the sources of financial or non-financial support and the roles of funders or sponsors in each of the versions of the LSR.**

Explanation
As with any research publication, transparency about the sources of financial or non-financial support is key. In the specific case of LSRs, it is important for readers of an LSR to be aware of financial or non-financial support for each of the LSR versions, as bias could have been introduced in an earlier version. Accordingly, this element highlights that LSRs should list all sources of support at any time. This element could be reported in the format of a table, with information for different versions displayed in different rows.

Examples
No examples found.

Competing interests

PRISMA 2020 item 26: Declare any competing interests of review authors.

- **PRISMA-LSR element: Describe the competing interests of review authors and how they were managed for each of the versions of the LSR.**

Explanation
As with any research publication, transparency about the competing interests of authors is key. In the specific case of LSRs, as authors are added or removed, and as competing interests might emerge or cease for existing authors, it is important for readers of an LSR to be aware of competing interests of authors for each of the LSR versions, as bias could have been introduced in an earlier version. Accordingly, this element highlights that LSRs should list all competing interests at any time. This element could be reported in the format of a table, with information for different versions displayed in different rows.

Examples
No examples found.

Availability of data, code and other materials

PRISMA 2020 item 27: Report which of the following are publicly available and where they can be found: template data collection forms; data extracted from included studies; data used for all analyses; analytic code; any other materials used in the review.

- **PRISMA-LSR element: Describe any changes since the preceding version to the accessibility of data, code and materials.**

Explanation
Describing any changes since the preceding version to the accessibility of data, code and materials can help readers easily determine where to locate that information. Refer to the Findable, Accessible, Interoperable and Reusable (FAIR) Guiding Principles for scientific data management and stewardship.[50]

Examples
No examples found.

Authors and their roles for each version of the LSR

PRISMA-LSR item L4: Provide the list of authors and their roles for each of the versions of the LSR.

Explanation
It is possible that the relative contributions of authors will vary across versions of an LSR, and that the author team will change across versions.[22] Indeed, in a mixed-methods evaluation with participants involved in Cochrane and non-Cochrane LSRs, participants discussed authorship issues as a complexity in the production of LSRs. Participants mentioned that the first versions of the reviews have a large authorship team while the subsequent versions with smaller changes required a much smaller team. The opportunity for contribution is further restricted by the speed of the updates. This led the author teams to question when people should come off the author list, and to request more guidance around this issue.[51] This element highlights that LSRs should list all authors and their roles at any time, acknowledging authors of previous versions who may not be authors of the current version. This element could be reported in the format of a table, with information for different versions displayed in different rows. Authors could have their competing interests listed in the same table. Refer to the CrediT (Contributor Roles Taxonomy) system for reporting on author roles.[52]

Example
"The list of authors has changed between the protocol and the first review version, and has also changed with each update version. Changes to the author list since the protocol to the current review version are outlined below."[29]

3. Explanation and examples for LSR status

Explanation
The LSR status refers to information published after the latest publication of an LSR version to indicate whether that LSR is ongoing or retired (ie, living status of the review). If it is ongoing, authors could also indicate whether any new studies have been identified, and whether they are being incorporated into the forthcoming version (ie, status of the evidence). While the PRISMA-LSR checklist applies to the report of a specific LSR version, the LSR status checklist does not. The LSR status checklist should be used to provide up-to-date information about the living status of the review as well as the status of the evidence in-between published LSR versions.

Examples

1. Reis S. et al. maintain an excel spreadsheet that is publicly accessible on the Open Science Framework (OSF) platform and that details the living evidence surveillance process that occurs between published LSR versions (see figure 2 below). Among other information, the authors highlight in the spreadsheet whether the LSR is ongoing or retired, by indicating if a new study changes the living mode of the review. They also indicate all of the LSR search dates (including the date of the last search), list of studies identified as eligible following each search, and whether a review update is in preparation. In addition, the project webpage on OSF indicates the date of its last update (ie, date the status was last updated).[1-3]
2. "As of September 12th, 2020, the team has stopped reviewing new references for research question 2 […]. Questions 1 and 3 […] will remain living systematic reviews. Since the project's beginning, question 2 has posed challenges, as it assesses findings from cross-sectional studies, not studies which evaluate changes over time […]. Additionally, the very high volume of low-quality studies eligible for question 2 has posed a challenge to the team's ability to keep evidence current. Since cross-sectional model-based studies are highly dependent on completeness and design of the models, many eligible studies have been at high risk of bias. This has negatively impacted our ability to highlight trends and report useful conclusions. Due to these concerns, including the rapidly expanding volume of potentially eligible studies for question 2 and our team's limited resources, we will analyze studies published as of September 12, 2020, but will not maintain a living systematic review for question 2 after that date."[53]

Date of last search	# records received						# records deduplicated	# records screened		Categorisation of reports included at fulltext screening								Trigger to publish update	Trigger to change living mode or stop updating	Review published incorporating this search	Date review published
	CCSR	Scopus	WHO	Total records from database search	Other sources	Total records database + other sources	Total after deduplication in EndNote	title/ abstract	fulltext	# included studies	# reports of included studies	# ongoing studies	# reports of ongoing studies	# studies awaiting classification	# reports of studies awaiting	# reports excluded	# additional manual deduplication				
11/04/2022	13	30	38	81	0	81	60	60	13	1	4	2	6	0	0	3	0	yes (EPIC-HR)	no	n/a	n/a
11/05/2022	69	41	51	161	0	161	74	74	7	0	0	4	4	0	0	3	0	no	no	n/a	n/a
10/06/2022	71	56	60	187	0	187	21	21	2	0	0	2	2	0	0	0	0	no	no	n/a	n/a
11/07/2022	78	68	71	217	0	217	22	22	1	0	0	0	0	0	0	1	0	no	no	https://www.cochranelibrary.com	20/09/2022
11/08/2022	81	91	87	259	0	259	27	27	6	0	0	0	0	0	0	6	0	no	no	n/a	n/a
12/09/2022	87	110	91	288	0	288	35	35	7	0	0	0	0	0	0	6	1	no	no	n/a	n/a
13/10/2022	98	133	104	335	0	335	32	32	12	0	0	3	3	0	0	9	0	no	no	n/a	n/a
11/11/2022	101	157	125	383	0	383	34	34	5	0	0	1	2	0	0	3	0	no	no	n/a	n/a
15/12/2022	106	184	139	429	0	429	41	41	6	0	0	2	2	1	1	3	0	no	no	n/a	n/a
18/01/2023	111	206	174	491	0	491	41	41	8	0	1	1	3	0	0	2	2	no	no	n/a	n/a
16/03/2023	146	280	191	617	1	618	113	113	12	1	4	-3	-5	0	1	12	0	yes (Liu-2023)	no	Review update in preparation	n/a
15/05/2023	165	333	241	739	-1	739	93	93	17	0	0	1	-2	2	6	13	1	no	no	n/a	n/a
21/07/2023	182	388	264	834	0	834	108	108	7	0	0	5	5	0	0	2	0	no	no	n/a	n/a
15/09/2023	212	464	264	940	0	940	102	102	6	0	0	-1	-1	0	0	6	0	no	no	n/a	
15/11/2023																					
Total	212	464	264	940	0	940	803	803	109	2	9	17	19	3	8	69	4				

Figure 2. Snapshot of excel spreadsheet detailing the living evidence surveillance process by Reis et al. [1-3]

4. Publishing issues

- Indication of whether the latest version has been peer reviewed. The rationale is that in standard SRs, peer review is standard. For LSRs, the initial version is typically peer-reviewed but the later versions might not be.
- Ability to include the version number in the title. This might depend on the publication process and platform used (as opposed to the reporting by the authors).
- Indication of whether the version being accessed by the readers is the latest version of the LSR, and if not, linking readers to the latest version.
- Indexing different versions of the same LSR in bibliographic databases.
- Provision of a stable link to access the LSR protocol; ie, citation, DOI, or permalink.
- Provision of a permanent link to files deposited in a stable public repository.
- Linking to older versions of data, code, and materials.

5. Conclusion

The PRISMA-LSR extension is intended to benefit LSR authors, editors and peer reviewers of LSRs, and different users of LSRs. This E&E document provides additional explanation on the application of the PRISMA-LSR extension's items and elements and should be used in conjunction with the PRISMA-LSR document. We hope that the implementation of the reporting guidance will improve transparency, completeness, and accuracy of LSR report.

References

1. Reis S, Metzendorf M-I, Kuehn R, et al. Nirmatrelvir combined with ritonavir for preventing and treating COVID-19. *Cochrane Database Syst Rev* 2022(9):CD015395.
2. Metzendorf M-I, Weibel S, Reis S, McDonald S. Pragmatic and open science-based solution to a current problem in the reporting of living systematic reviews. *BMJ Evidence-Based Medicine* 2023;28(4):267-72.
3. Reis S, Metzendorf M-I, Kuehn R, et al. Living evidence base for Cochrane Review “Nirmatrelvir combined with ritonavir for preventing and treating COVID-19”: OSF; 2023. Available from: osf.io/7g49c.
4. Welch V, Petticrew M, Petkovic J, et al. Extending the PRISMA statement to equity-focused systematic reviews (PRISMA-E 2012): explanation and elaboration. *J Clin Epidemiol* 2016;70:68-89.
5. Page MJ, Moher D, Bossuyt PM, et al. PRISMA 2020 explanation and elaboration: updated guidance and exemplars for reporting systematic reviews. *BMJ* 2021;372:n160
6. Cohen JF, Deeks JJ, Hooft L, et al. Preferred reporting items for journal and conference abstracts of systematic reviews and meta-analyses of diagnostic test accuracy studies (PRISMA-DTA for Abstracts): checklist, explanation, and elaboration. *BMJ* 2021;372:n265
7. Hutton B, Salanti G, Caldwell DM, et al. The PRISMA extension statement for reporting of systematic reviews incorporating network meta-analyses of health care interventions: checklist and explanations. *Ann Intern Med* 2015;162(11):777-84.
8. Salameh J-P, Bossuyt PM, McGrath TA, et al. Preferred reporting items for systematic review and meta-analysis of diagnostic test accuracy studies (PRISMA-DTA): explanation, elaboration, and checklist. *BMJ* 2020;370:m2632
9. Ghosn L, Chaimani A, Evrenoglou T, et al. Interleukin-6 blocking agents for treating COVID-19: a living systematic review. *Cochrane Database Syst Rev* 2021(3):CD013881.
10. Siemieniuk RA, Bartoszko JJ, Ge L, et al. Drug treatments for covid-19: living systematic review and network meta-analysis. *BMJ* 2020;370:m2980
11. Moher D, Shamseer L, Clarke M, et al. Preferred reporting items for systematic review and meta-analysis protocols (PRISMA-P) 2015 statement. *Syst Rev* 2015;4:1-9.
12. Counotte MJ, Meili KW, Taghavi K, et al. Zika virus infection as a cause of congenital brain abnormalities and Guillain-Barré syndrome: A living systematic review [version 1; peer review: 2 approved]. *F1000Research* 2019;8:1433.
13. Xu W, Li X, Dong Y, et al. SARS-CoV-2 transmission in schools: An updated living systematic review (version 2; November 2020). *Journal of Global Health* 2021;11:10004.
14. Kaka AS, MacDonald R, Linskens EJ, et al. Major update 2: remdesivir for adults with COVID-19: a living systematic review and meta-analysis for the American College of Physicians practice points. *Ann Intern Med* 2022;175(5):701-09.
15. Elliott JH, Synnot A, Turner T, et al. Living systematic review: 1. Introduction—the why, what, when, and how. *J Clin Epidemiol* 2017;91:23-30.
16. Counotte MJ, Kim CR, Wang J, et al. Sexual transmission of Zika virus and other flaviviruses: a living systematic review. *PLoS Med* 2018;15(7):e1002611.
17. Drucker AM, Ellis A, Jabbar-Lopez Z, et al. Systemic immunomodulatory treatments for atopic dermatitis: protocol for a systematic review with network meta-analysis. *BMJ Open* 2018;8(8):e023061.
18. Lawrenson JG, Shah R, Huntjens B, et al. Interventions for myopia control in children: a living systematic review and network meta-analysis. *Cochrane Database Syst Rev* 2023(2):CD014758.
19. Kahale LA, Elkhoury R, El Mikati I, et al. Tailored PRISMA 2020 flow diagrams for living systematic reviews: a methodological survey and a proposal [version 3; peer review: 2 approved]. *F1000Research* 2021;10:192.

20. Iannizzi C, Dorando E, Burns J, et al. Methodological challenges for living systematic reviews conducted during the COVID-19 pandemic: A concept paper. *J Clin Epidemiol* 2022;141:82-89.
21. Iannizzi C, Chai KL, Piechotta V, et al. Convalescent plasma for people with COVID-19: a living systematic review. *Cochrane Database Syst Rev* 2023(2):CD013600.
22. Cochrane Collaboration. Guidance for the production and publication of Cochrane living systematic reviews: Cochrane Reviews in living mode. Version December 2019. Available from: https://community.cochrane.org/sites/default/files/uploads/inline-files/Transform/201912_LSR_Revised_Guidance.pdf.
23. Akl EA, El Khoury R, Khamis AM, et al. The life and death of living systematic reviews: a methodological survey. *J Clin Epidemiol* 2023;156:11-21.
24. Iannizzi C, Akl EA, Anslinger E, et al. Methods and guidance on conducting, reporting, publishing and appraising living systematic reviews: a scoping review. *Syst Rev* 2023;12(1):238.
25. Boutron I, Chaimani A, Devane D, et al. Interventions for the treatment of COVID-19: a living network meta-analysis. *Cochrane Database Syst Rev* 2020(11):CD013770.
26. Murad MH, Wang Z, Chu H, et al. Proposed triggers for retiring a living systematic review. *BMJ Evidence-Based Medicine* 2023;28(5):348-52.
27. Wilt TJ, Kaka AS, MacDonald R, et al. Remdesivir for adults with COVID-19: a living systematic review for American College of Physicians practice points. *Ann Intern Med* 2021;174(2):209-20.
28. Bero L, Lawrence R, Leslie L, et al. Cross-sectional study of preprints and final journal publications from COVID-19 studies: discrepancies in results reporting and spin in interpretation. *BMJ Open* 2021;11(7):e051821.
29. Ebrahimzadeh S, Islam N, Dawit H, et al. Thoracic imaging tests for the diagnosis of COVID-19. *Cochrane Database Syst Rev* 2022(5):CD013639.
30. Siemieniuk RA, Bartoszko JJ, Martinez JPD, et al. Antibody and cellular therapies for treatment of covid-19: a living systematic review and network meta-analysis. *BMJ* 2021;374:n2231
31. Juul S, Nielsen EE, Feinberg J, et al. Interventions for treatment of COVID-19: A living systematic review with meta-analyses and trial sequential analyses (The LIVING Project). *PLoS Med* 2020;17(9):e1003293.
32. Simmonds M, Salanti G, McKenzie J, et al. Living systematic reviews: 3. Statistical methods for updating meta-analyses. *J Clin Epidemiol* 2017;91:38-46.
33. Elliott J, DeJean D, Clifford T, et al. Cannabis for pediatric epilepsy: protocol for a living systematic review. *Syst Rev* 2018;7:1-5.
34. Rethlefsen ML, Kirtley S, Waffenschmidt S, et al. PRISMA-S: an extension to the PRISMA statement for reporting literature searches in systematic reviews. *Syst Rev* 2021;10:1-19.
35. Buitrago-Garcia D, Ipekci AM, Heron L, et al. Occurrence and transmission potential of asymptomatic and presymptomatic SARS-CoV-2 infections: update of a living systematic review and meta-analysis. *PLoS Med* 2022;19(5):e1003987.
36. Haddaway NR. livingPRISMAflow: R package and ShinyApp for producing living PRISMA flow diagrams (Version 0.0.1) zenodo 2021. Available from: https://doi.org/10.5281/zenodo.4572459.
37. Ute Muti-Schüenemann GE, Szczeklik W, Solo K, et al. Update Alert 3: Ventilation Techniques and Risk for Transmission of Coronavirus Disease, Including COVID-19. *Ann Intern Med* 2022;175(1):W6-W7.
38. Juul S, Nielsen EE, Feinberg J, et al. Interventions for treatment of COVID-19: Second edition of a living systematic review with meta-analyses and trial sequential analyses (The LIVING Project). *PLoS One* 2021;16(3):e0248132.
39. Reis S, Metzendorf M-I, Kuehn R, et al. Nirmatrelvir combined with ritonavir for preventing and treating COVID-19. *Cochrane Database Syst Rev* 2023(11):CD015395.
40. Bartoszko JJ, Siemieniuk RA, Kum E, et al. Prophylaxis against covid-19: living systematic review and network meta-analysis. *BMJ* 2021;373:n949
41. Woodcock J, LaVange LM. Master protocols to study multiple therapies, multiple diseases, or both. *N Engl J Med* 2017;377(1):62-70.

42. Santesso N, Carrasco-Labra A, Langendam M, et al. Improving GRADE evidence tables part 3: detailed guidance for explanatory footnotes supports creating and understanding GRADE certainty in the evidence judgments. *J Clin Epidemiol* 2016;74:28-39.
43. Hernandez AV, Roman YM, Pasupuleti V, Barboza JJ, White CM. Update alert: hydroxychloroquine or chloroquine for the treatment or prophylaxis of COVID-19. *Ann Intern Med* 2020;173(4):W78-W79.
44. Kansagara D, Mackey K, Vela K. Update alert: risks and impact of angiotensin-converting enzyme inhibitors or angiotensin-receptor blockers on SARS-CoV-2 infection in adults. *Ann Intern Med* 2020;173(3):W66.
45. Hernandez AV, Roman YM, Pasupuleti V, Barboza JJ, White CM. Update alert 3: hydroxychloroquine or chloroquine for the treatment or prophylaxis of COVID-19. *Ann Intern Med* 2020;173(11):W156-W57.
46. Kaka AS, MacDonald R, Greer N, et al. Major update: remdesivir for adults with COVID-19: a living systematic review and meta-analysis for the American College of Physicians practice points. *Ann Intern Med* 2021;174(5):663-72.
47. John A, Eyles E, Webb RT, et al. The impact of the COVID-19 pandemic on self-harm and suicidal behaviour: update of living systematic review [version 2; peer review: 1 approved, 2 approved with reservations]. *F1000Research* 2020;9:1097.
48. Ferrara R, Imbimbo M, Malouf R, et al. Single or combined immune checkpoint inhibitors compared to first-line platinum-based chemotherapy with or without bevacizumab for people with advanced non-small cell lung cancer. *Cochrane Database Syst Rev* 2020(12):CD013257.
49. Spurling GK, Del Mar CB, Dooley L, Clark J, Askew DA. Delayed antibiotic prescriptions for respiratory infections. *Cochrane Database Syst Rev* 2017(9):CD004417.
50. Wilkinson MD, Dumontier M, Aalbersberg IJ, et al. The FAIR Guiding Principles for scientific data management and stewardship. *Scientific data* 2016;3(1):1-9.
51. Millard T, Synnot A, Elliott J, et al. Feasibility and acceptability of living systematic reviews: results from a mixed-methods evaluation. *Syst Rev* 2019;8:1-14.
52. Allen L, Scott J, Brand A, Hlava M, Altman M. Publishing: Credit where credit is due. *Nature* 2014;508(7496):312-13.
53. Living systematic review of mental health in COVID-19. Available from: https://www.depressd.ca/covid-19-mental-health.
