# Supplementary material for: Extension of the PRISMA 2020 statement for living systematic reviews (PRISMA-LSR): checklist and explanation
Source: BMJ. 2024 Nov 19;387:e079183. doi: 10.1136/bmj-2024-079183 (PMC12036629; doi:10.1136/bmj-2024-079183)

Figure S1: LSR-tailored flow diagram (approach 1)

Identification of new studies via databases and registers

Identification

Records identified from:

Databases

Jan 2020	Feb 2020	Mar 2020	Apr 2020	May 2020	Jun 2020	Jul 2020	Aug 2020
90	0	2	8	19	0	2	4

Registers

10	1	4	1	0	0	8	1

Records removed before screening

Duplicates

Jan 2020	Feb 2020	Mar 2020	Apr 2020	May 2020	Jun 2020	Jul 2020	Aug 2020
0	0	1	3	0	0	2	0

Automation

10	0	0	1	1	0	0	0

Other

0	0	0	2	0	0	0	0

Screening

Records screened

Jan 2020	Feb 2020	Mar 2020	Apr 2020	May 2020	Jun 2020	Jul 2020	Aug 2020
90	1	5	3	18	0	8	5

Records excluded

Jan 2020	Feb 2020	Mar 2020	Apr 2020	May 2020	Jun 2020	Jul 2020	Aug 2020
82	1	5	3	17	0	8	4

Reports sought for retrieval

Jan 2020	Feb 2020	Mar 2020	Apr 2020	May 2020	Jun 2020	Jul 2020	Aug 2020
8	0	0	0	1	0	0	1

Reports not retrieved

Jan 2020	Feb 2020	Mar 2020	Apr 2020	May 2020	Jun 2020	Jul 2020	Aug 2020
0	0	0	0	0	0	0	0

Reports assessed for eligibility

Jan 2020	Feb 2020	Mar 2020	Apr 2020	May 2020	Jun 2020	Jul 2020	Aug 2020
8	0	0	0	1	0	0	1

Reports excluded

Jan 2020	Feb 2020	Mar 2020	Apr 2020	May 2020	Jun 2020	Jul 2020	Aug 2020
3	0	0	0	0	0	0	1

Not intervention of interest, 1 (Aug 2020)
Not intervention of interest, 1 (Jan 2020)
Not population of interest, 2 (Jan 2020)

Included

New studies included in review

Jan 2020	Feb 2020	Mar 2020	Apr 2020	May 2020	Jun 2020	Jul 2020	Aug 2020
5	0	0	1	1	0	0	0

New reports included in review

9	0	0	1	1	0	0	0

Identification of new studies via other methods

Records identified from:

Websites

Jan 2020	Feb 2020	Mar 2020	Apr 2020	May 2020	Jun 2020	Jul 2020	Aug 2020
0	0	1	1	0	0	0	0

Organisations

7	0	0	0	0	0	0	0

Citations

0	0	0	0	0	0	0	0

Reports sought for retrieval

Jan 2020	Feb 2020	Mar 2020	Apr 2020	May 2020	Jun 2020	Jul 2020	Aug 2020
7	0	1	1	0	0	0	0

Reports not retrieved

Jan 2020	Feb 2020	Mar 2020	Apr 2020	May 2020	Jun 2020	Jul 2020	Aug 2020
1	0	1	0	0	0	0	0

Reports assessed for eligibility

Jan 2020	Feb 2020	Mar 2020	Apr 2020	May 2020	Jun 2020	Jul 2020	Aug 2020
6	0	0	1	0	0	0	0

Reports excluded

Jan 2020	Feb 2020	Mar 2020	Apr 2020	May 2020	Jun 2020	Jul 2020	Aug 2020
2	0	0	0	0	0	0	0

Not design of interest, 2 (Jan 2020)

Figure S2: LSR-tailored flow diagram (approach 2)

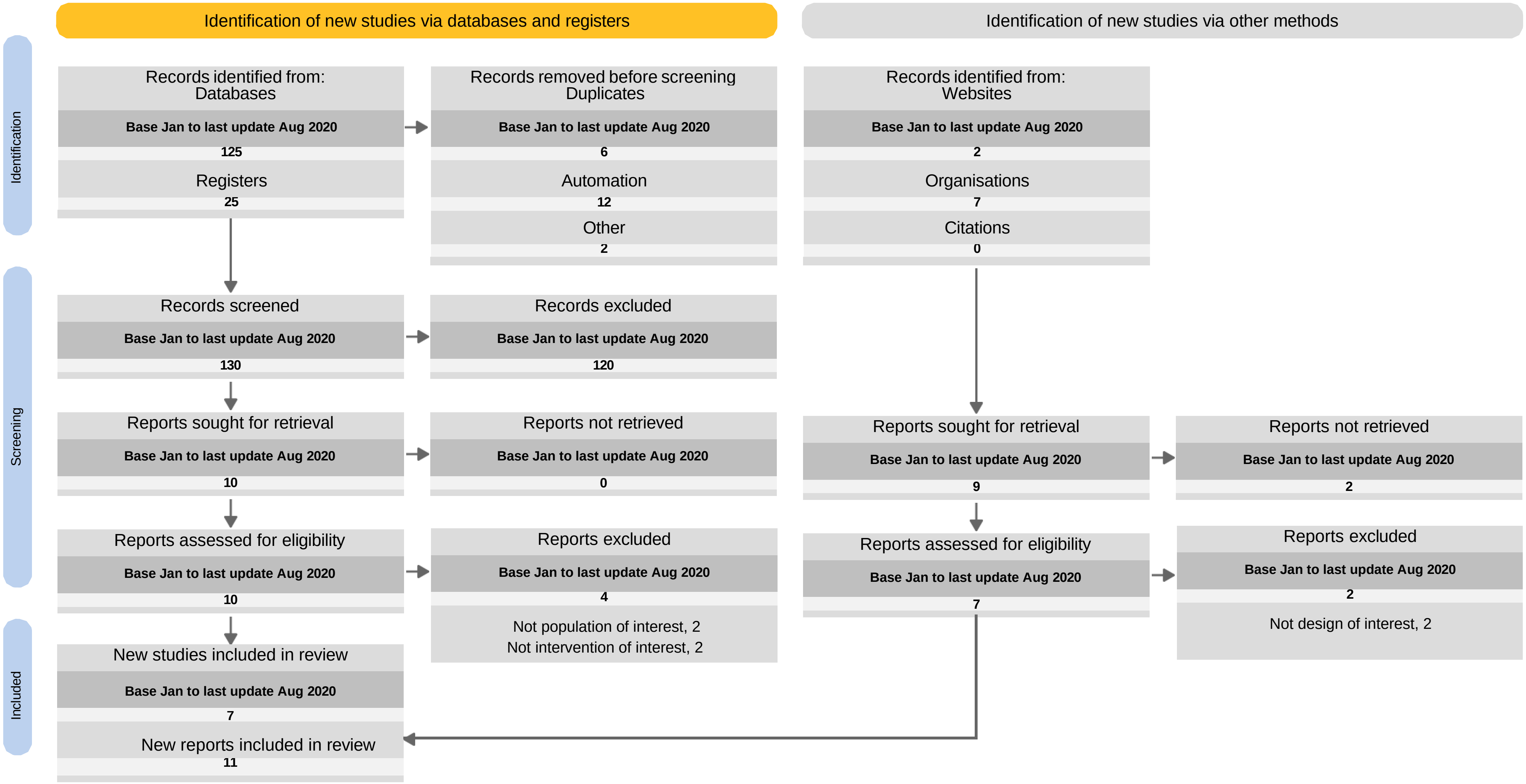

Figure S3: LSR-tailored flow diagram (approach 3)

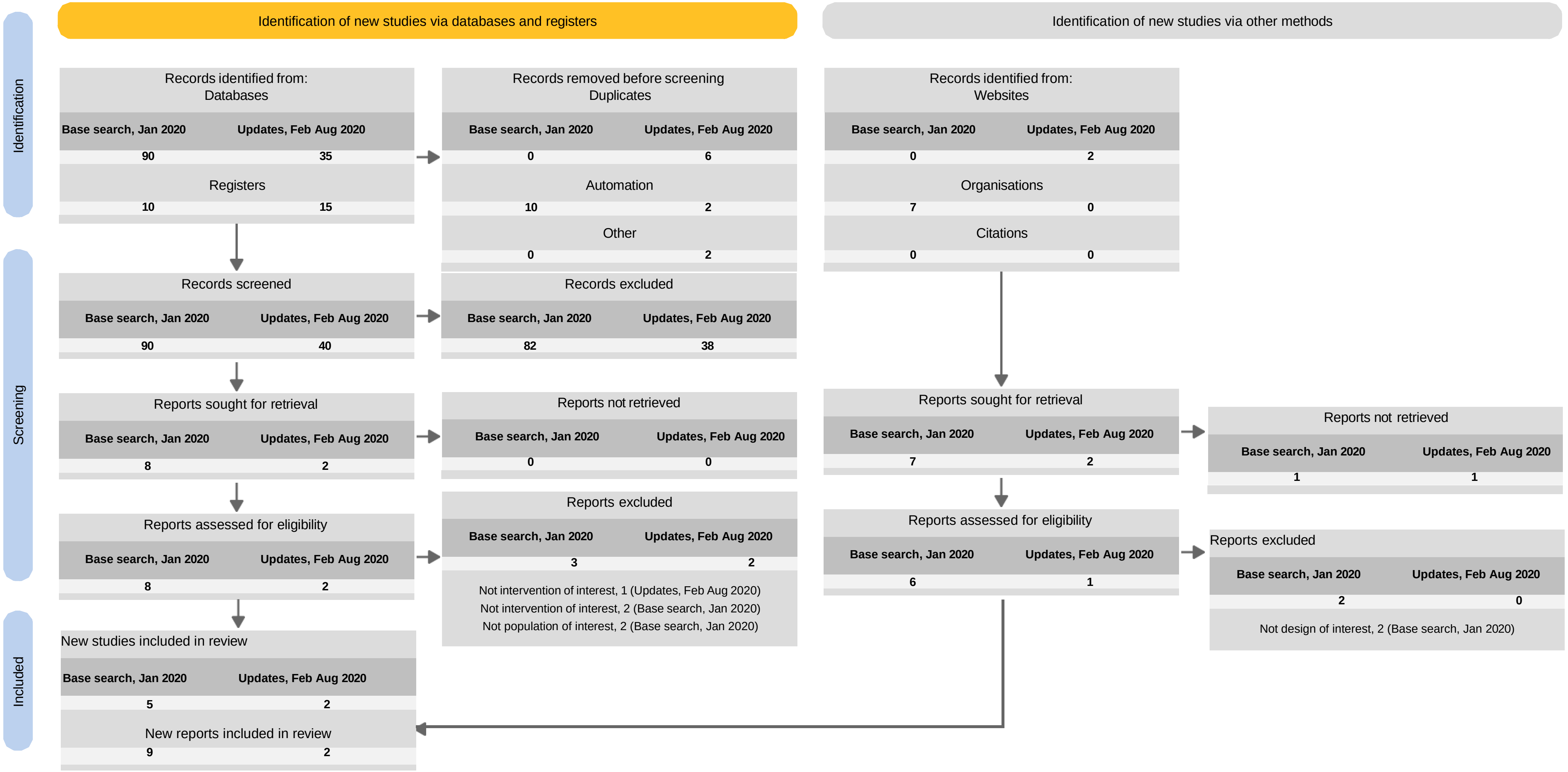

Figure S4: LSR-tailored flow diagram (approach 4)

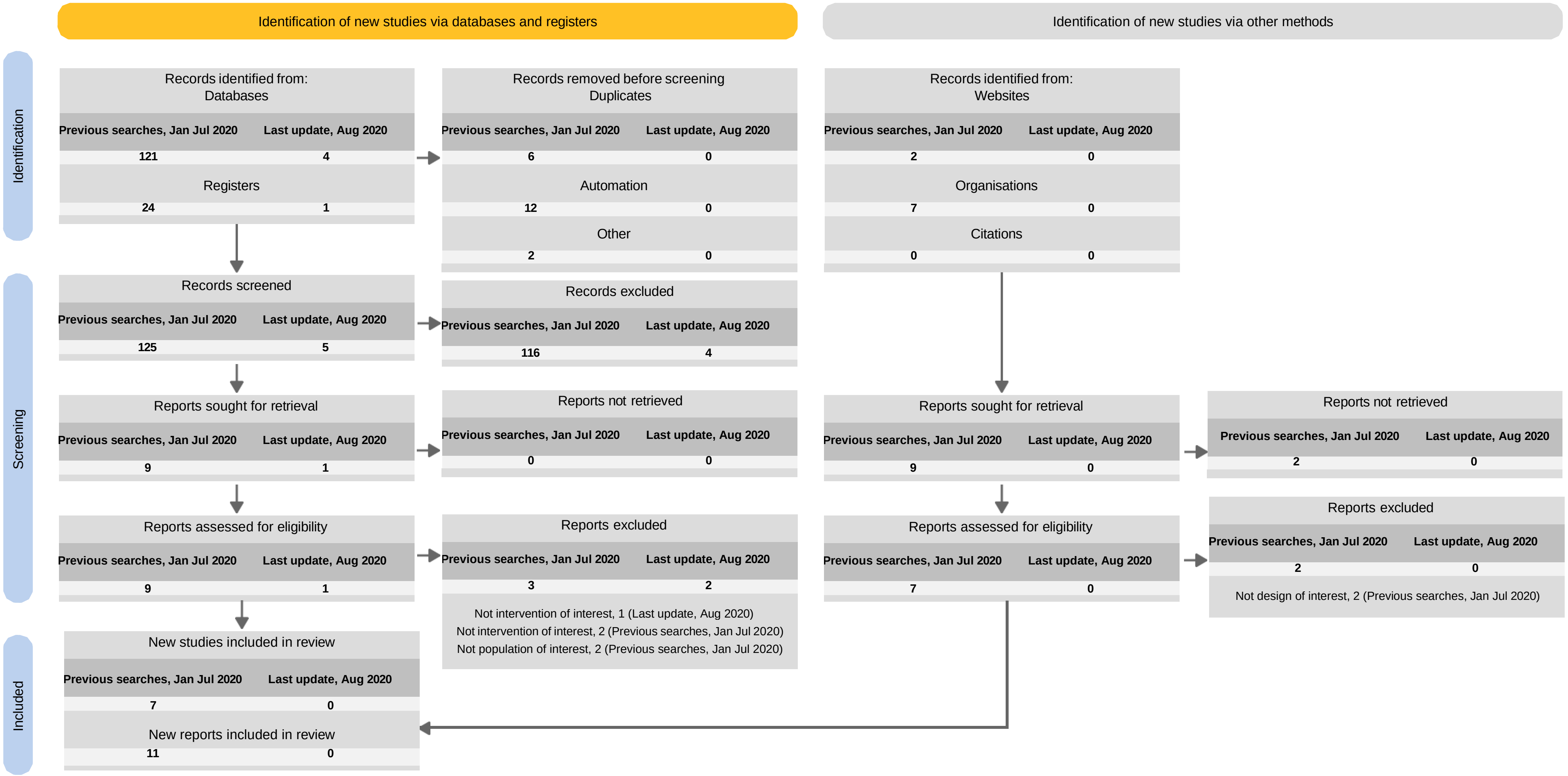

Supplement: Supplementary file 4 — Web appendix 4: Figures S1-S4 [file akle079183.ww4.pdf]
